# Genome-Wide Transcriptome Landscape of Embryonic Brain-Derived Neural Stem Cells Exposed to Alcohol with Strain-Specific Cross-Examination in BL6 and CD1 Mice

**DOI:** 10.1038/s41598-018-36059-y

**Published:** 2019-01-18

**Authors:** Wayne Xu, Vichithra R. B. Liyanage, Aaron MacAulay, Romina D. Levy, Kyle Curtis, Carl O. Olson, Robby M. Zachariah, Shayan Amiri, Marjorie Buist, Geoffrey G. Hicks, James R. Davie, Mojgan Rastegar

**Affiliations:** 1 Department of Biochemistry and Medical Genetics, Max Rady College of Medicine, Rady Faculty of Health Sciences, University of Manitoba, Winnipeg, Canada; 2 Research Institute of Oncology and Hematology, CancerCare Manitoba, Winnipeg, Canada; 3 Regenerative Medicine Program, Max Rady College of Medicine, Rady Faculty of Health Sciences, University of Manitoba, Winnipeg, Canada

## Abstract

We have previously reported the deregulatory impact of ethanol on global DNA methylation of brain-derived neural stem cells (NSC). Here, we conducted a genome-wide RNA-seq analysis in differentiating NSC exposed to different modes of ethanol exposure. RNA-seq results showed distinct gene expression patterns and canonical pathways induced by ethanol exposure and withdrawal. Short-term ethanol exposure caused abnormal up-regulation of synaptic pathways, while continuous ethanol treatment profoundly affected brain cells’ morphology. Ethanol withdrawal restored the gene expression profile of differentiating NSC without rescuing impaired expression of epigenetics factors. Ingenuity Pathway Analysis (IPA) analysis predicated that ethanol may impact synaptic functions *via* GABA receptor signalling pathway and affects neural system and brain morphology. We identified *Sptbn2*, *Dcc*, and *Scn3a* as candidate genes which may link alcohol-induced neuronal morphology to brain structural abnormalities, predicted by IPA analysis. Cross-examination of *Scn3a* and *As3mt* in differentiated NSC from two different mouse strains (BL6 and CD1) showed a consistent pattern of induction and reduction, respectively. Collectively, our study identifies genetic networks, which may contribute to alcohol-mediated cellular and brain structural dysmorphology, contributing to our knowledge of alcohol-mediated damage to central nervous system, paving the path for better understanding of FASD pathobiology.

## Introduction

Fetal Alcohol Spectrum Disorders (FASD) refer to a variety of neurodevelopmental abnormalities due to prenatal alcohol (ethanol) exposure (PAE). In a representative Midwestern community in the United States, FASD rate amongst grade one students (average age of 6–7 years) is estimated at 2.4–4.8%^1^. In Canada, FASD prevalence rate is estimated at 1%, with higher rates reported in Alberta (up to 4.8%)^2^. FASD etiology is thought to be through alcohol cytotoxicity and *in utero* alcohol-gene interactions as well as deregulated cellular gene expression programs during brain development. The outcome of PAE may vary from a broad spectrum of embryonic death to birth defects, functional abnormalities of FASD patients with intellectual disability, and aberrant behavior later in life^3^.

Intensive efforts have focused on discovering FASD mechanism of disease to provide explanations on how alcohol affects fetal development. Once alcohol enters the cell, it is metabolized through specific cellular pathways. In the cytoplasm, alcohol dehydrogenase facilitates the change of ethanol to acetaldehyde, which subsequently interacts with other biomolecules, including genomic DNA and cellular proteins. One major damaging outcome of prenatal alcohol exposure is transcriptional abnormality in developing brain cells that will ultimately determine future phenotypes and FASD symptoms^3^. To study the impact of alcohol on the developing brain, primary culture of brain cells and neural stem cells (NSC) are extensively used^4–7^. In different model systems, the impact of alcohol is reported on the Sonic Hedgehog pathway^8^, cell signaling molecules^9^, gene copy number variants^10^, and genes involved in alcohol metabolism^11^. In embryonic NSC, alcohol deregulated several proteins including structural proteins^4^.

Epigenetics play key roles in ethanol-induced change in gene expression of brain cells^3^. Ethanol can modify epigenetic mechanisms either altering DNA modifications and/or post-translational modification of histone proteins^12^. To study the impact of ethanol on differentiated NSC, a range of different ethanol concentrations up to 100 mM is commonly used^4^, with 22–69 mM ethanol reported to have minimal effects on NSC differentiation^7^. In embryonic NSC exposed to 70 mM ethanol for 8 days, we showed that differentiation is not affected, but ethanol induces 5mC levels, while 2 days ethanol exposure does not alter 5mC/5hmC levels. Conversely, differentiating NSC retained memory of short-term ethanol exposure by decreased levels of 5hmC (not 5mC), when ethanol was removed for 6 days. This study showed that short- and long-term ethanol exposure also induces the expression of DNA methyl binding protein “MeCP2”, while ethanol withdrawal reduces it^13^. This study showed that short- and long-term ethanol exposure (or withdrawal) might differentially impact DNA methylation^13^. Differentiating NSC are proven useful systems not only to study developmentally important genes, but also for modeling therapy strategies of neurodevelopmental disorders^14–19^.

In this current study, we applied a genome-wide transcriptome analysis strategy to explore the genetic networks that are associated with altered gene expression program of brain-derived NSC under short-term and long-term ethanol exposure, compared to ethanol withdrawal. Alteration of these identified gene networks may ultimately contribute to abnormal brain morphology upon ethanol exposure.

## Materials and Methods

### Ethics statement

All the conducted experiments were done in accordance with standards and guidelines of the Canadian Council on Animal Care. All procedures were reviewed and approved by Office of Research Ethics of University of Manitoba, and under the animal protocol number 12-031/1/2/3 (AC10709).

### Isolation, culture conditions, and differentiation of brain-derived neural stem cells

Isolation of neural stem cells from the C57BL/6 (BL6) or CD1 murine forebrains at E14.5 and culture condition were the same as we previously described^13–16^. Briefly, we dissected the forebrain tissues and homogenized tissue pieces in NSC culture media, by addition of EGF and FGF to basic NSC media as described in detail in our previous reports^13–16^. We cultured cells for 7 days for primary neurospheres. Dissociated neurospheres were differentiated in the presence of FBS, and removal of EGF/FGF for 8 days as we have shown is sufficient for the differentiation of neuronal and glial cells^13–16^.

### Ethanol treatment

Ethanol treatments were done as previously reported in a recent study from our group^13^. Briefly, we dissociated the neurosphere cells and added ethanol at day (D)0 (start of cell differentiation) with ethanol (Commercial Alcohols) at a final concentration of 70 mM. Ethanol concentration was done based on previous research, (a) reporting conditions which alters neuronal and glial morphology without altering NSC cell fate^13^, (b) previously reported blood alcohol level that is relevant for alcoholics (30–100 mM)^20^, (c) reported deregulatory role of ethanol on human neural stem cells from fetal brain (20–100 mM)^21^, and lastly (d) established conditions with minimal effect on NSC division and culture (22–70 mM)^7,13^. For short-term ethanol treatment (binge), we only added ethanol once at the start of the differentiation culture at D0 and for 48 h. We collected the cells at D2. For long-term ethanol treatment (continuous), we exposed the cells to ethanol from D0 for 8 days with media change and new ethanol being added every two days. For ethanol withdrawal, we changed the culture media after 48 h from initial addition of ethanol at D0, and no ethanol was added after D2 (Supplementary Fig. S1). We cultured the differentiating NSC for an extra 6 days and refreshed media every two days. We cultured control NSC with similar conditions without ethanol. Cells were harvested at D0, D2, and D8.

### RNA library preparation, and high throughput RNA-sequencing (RNA-seq)

Total RNA from differentiating neural stem cells (D2 or D8) under different conditions was isolated by the RNeasy Mini Kit (Qiagen). RNA-seq was carried out as a paid service from the McGill University and Génome Québec Innovation Centre, Canada. Total RNA was first checked for quality control and determining the purity of samples using the Agilent Bioanalyzer 2100 (with ribosomal RNA depletion). The cDNA libraries were prepared using first-strand “TrueSeq mRNA” protocol. RNA samples were then sequenced by Illumina platform.

### qRT-PCR validation

RNA-seq validation experiments were performed on the RNA sample triplicates that we used for RNA-seq studies. An additional three sets of RNA samples from CD1 murine strain were prepared and subjected to RT-PCR analysis for cross-examination of selected genes in CD1 NSC by RT-PCR. We prepared cDNA from the total RNA with Invitrogen Superscript III Reverse Transcriptase as described earlier^15,22–24^. QRT-PCR was done with SYBR Green-based RT^2^ qPCR Master Mix and in an Applied Biosystems Fast 7500 Real-Time PCR machine according to the details that are reported elsewhere^14,15,25^. For selected subset of genes, we performed RT-PCR and Ct values (threshold cycle) for the tested genes were normalized against a housekeeping gene (*Gapdh)*. We calculated the ∆Ct values for each specific gene and the relative expression analysis was done by Microsoft Excel 2010, comparing 2^−∆Ct^ of each specific sample to controls at D2 and D8 as we explained previously^15,25^. To confirm the specificity of the primers, PCR products were purified and sequenced at the MICB sequencing facility (Winnipeg, Canada) and Eurofins MWG Operon. The sequences of the primers used for RT-PCR are shown in Supplementary Table S1. RNA samples used for developmental and brain region-specific expression were the same as already reported by our team^26^. RNA samples to study the basal expression levels of *Mecp2* and other DNA methylation-related genes in BL6 and CD1 were the same as already reported by our team^14,15^.

### DNA dot blot and pyrosequencing at the *Mecp2* regulatory regions (R1-to-R6)

DNA was extracted from the forebrain of CD1 and BL6 mice by DNeasy Blood and Tissue kit (Qiagen) according to the manufacturer’s instructions. Collected DNA was subjected to dot blot experiments with a previously described protocol^13,15^, that consisted of DNA heat denaturation in 0.4 M sodium hydroxide (NaOH), 10 mM ethylenedi-aminetetraacetic acid (EDTA), neutralization with ice-cold ammonium acetate (2 M, pH 7.0), and UV DNA cross-linking on Zeta-Probe GT blotting membrane (Bio Rad). The membranes were blocked in 3–5% skim milk dissolved in PBST (PBS + 0.1% Tween), and primary incubation in primary antibodies [5mC, 1:1000 (Abcam, Ontario, Toronto, Canada, Ab73938) or 5hmC, 1:1000 (Active Motif, 39769) in PBST], followed by three standard washes, and incubation with secondary HRP-conjugated antibodies, and repeat of three standard washes. The signals were visualized by enhanced chemiluminescence, and total DNA levels were visualized by 0.02% methylene blue (MB) in 0.3 M sodium acetate (pH 5.2), and quantification of dot blot signals by Adobe Photoshop. To study DNA methylation at the *Mecp2* regulatory regions (R1-to-R6), collected DNA was submitted to the “Hospital for Sick Children (SickKids), Toronto, Canada”, and bisulfite pyrosequencing experiments were done as a paid service. The sequence of the primers for bisulfite pyrosequencing is already reported in our previous studies^13,15,25^. Correlational studies of DNA methylation with *Mecp2e1* and *Mecp2e2* was performed as previously reported^13,15,25^.

### RNA-seq data analysis

A total of approximately 2664 million sequence reads (paired 100 bp) of Illumina Hiseq were generated for 18 NSC samples at 6 conditions (average 148 million sequence reads per sample), each with 3 biological replicates from each conditions at D0, D2 for control, D8 for control, short-term ethanol treatment (Binge), long-term ethanol treatment (continuous) along with ethanol withdrawal. The quality check and quality trimming were applied to the sequence fastq files. We mapped the sequence reads on murine reference genome (mm10) using Tophat2.0 with default settings. An average of 88% of reads were precisely paired mapped (Supplementary Table S2). The mapped reads were quantified against the Ensembl annotation (Mus_musculus.GRCm38.75) using Cufflinks (v2.1.1). The mapped raw read counts, and FPKM were extracted from Cufflinks output files.

### Differential expression genes (DEGs) analysis

We used Bioconductor package edgeR according to Negative binomial model to calculate differential expression gene (DEG) between the ethanol-treated and control samples. The common dispersion and the value of variation of biological variation (BCV) of sample group were estimated using raw read count data from Cufflinks output and the Fisher’s Exact test was applied for significance test. The false discovery rate (FDR < 0.05) was a cut-off for significantly expressed genes.

### Gene set enrichment analysis (GSEA)

We downloaded the newest version of molecular signatures database (MSigDB v6.1) from http://software.broadinstitute.org/gsea/msigdb/. This database contains Gene Ontology terms for gene set enrichment analysis using human genes input. To perform GSEA for mouse gene list, we generated the mouse orthologs to human genes using ensemble v9.1 annotations. Since there were a few empty entries in FPKM data, we used the RPKM computed from raw read counts for GSEA analysis. 1000 permutations were performed against gene sets using the default signal-to-noise metric to rank the genes. The false discovery rate of FDR < 0.1 was a cut-off and the normalized enrichment scores was used for generating heatmap.

### Ingenuity Pathway Analysis

To investigate biological functions, the functional gene networks, the canonical pathways and disease conditions, differentially expressed gene sets were analyzed by IPA software (Ingenuity Pathways Analysis, Redwood City, CA, USA; http://www.ingenuity.com/products/ipa)^27^. We inputted gene lists into IPA that were differentially expressed with FDR < 0.05 and selected the log2 fold changes as color display. The direct and indirect interaction with the default setting of size of 35 entities among the Ingenuity curated database was performed for network analysis. The networks with enrichment score greater than 35 were selected for further analysis.

### Transcriptome profile and clustering

Expression, clustering and genomic feature visualization were done by using the Partek Genomics Suite v6.6 (Partek Inc. St Louis, USA). Transcriptome landscape and gene differential expression among all chromosomes were displayed using Circos software (http://circos.ca/).

### Immunocytochemistry (ICC) and immunohistochemical (IHC) studies

We used a previously established protocol to perform ICC for differentiating neural stem cells^15,16,26^. Briefly, cells were fixed in 4% formaldehyde, permeabilized with 2% NP40-PBS, and blocked with 10% NGS (normal goat serum) (Jackson Immunoresearch). Incubation with primary antibodies was overnight at 4 °C, and incubation with secondary antibodies was done at room temperature for 1 hour (h). Samples were then mounted on glass slides with antifade with added DAPI 2(μg/ml). Axio Observer Z1 inverted microscope was used to acquire the immunofluorescent signals and the images were obtained and analyzed using Zen 2012 software and made by Adobe Photoshop and Adobe Illustrator. Images were taken at the same exposure time for controls and treated samples. Cultured neurospheres at D0 were fixed with paraformaldehyde, sectioned, air-dried, and processed for IHC analysis, as we reported^14^. Briefly, samples on air-dried slides were permeabilized and pre-blocked with 0.3% Triton X-100 Tris-buffered saline, in 10% NGS. Primary antibody incubation in 10% NGS was done overnight at 4 °C, subsequent washes, secondary antibody incubation for 1 h, and standard IHC washes as reported. Then coverslips were slide mounted with glycerol anti-fade medium containing DAPI counter stain (0.5 μg/ml). The following antibodies were used for IHC studies: mouse rabbit anti-SOX2 (Millipore, Temecular CA, 1:250), rabbit anti-OLIG2 (Millipore, 1:1500), rabbit anti-MeCP2 (Millipore, 07–013), and rabbit anti-Ki67 (Santa cruz, sc-15402). Immunofluorescence signals were detected by Axio Observer Z1 inverted microscope and LSM710 Confocal microscope (Carl Zeiss, Canada Ltd, Toronto, ON), and images were processed by AxioVision 4.8, Zen Black 2011 softwares (Carl Zeiss).

### Quantification of neurite branching and measurements of glial cell size

The ImageJ program was used to quantify the number of neurites as described in our previous studies^13,16^. The images were adjusted to visualize all neurites. From each biological replicate, at least 20 TUB^+^ neurons were imaged. Neurons that exhibited no neurites or had unclear neurites  were eliminated from quantification. The longest neurite was assumed to be the axon^28^ and thus the length of the longest primary neurite was quantified using Zen program (Closed Bezier tool) which provided the length in µm. As an indication of glial cell size, the images were obtained at 0.32 µm sections (Z-stacks) were superimposed and then we estimated the surface area for GFAP^+^ astrocytes by spline contour tool from the Zen 2012 software similar to previous reports^13,29^. After adjusting the signal intensity to clearly observe outer margin of GFAP^+^ cells, the spline contour tool was used to carefully mark the outer margin of each cell. From each biological replicate, at least 20 GFAP^+^ astrocytes were measured.

### Statistical Analysis

Three different statistics were applied to the differential expression gene analysis in this study. The Cuffdiff module in Cufflinks software calculated the statistical significance of two groups using the three biological replicates (FPKM). Instead of using three replicate values, the Bioconductor edgeR package applied the negative binomial distribution of all reads to calculate the DEGs and significances, which provides more sensitivity than Cufflinks. For qRT-PCR, we made the graphs by Graphpad prism software with the average of 3 independent biological samples. The error bars show standard error of mean (SEM). We used Student’s *t*-test for statistical significance to report gene expression, neurite numbers and glial cell size. We show the statistical significance with *p < 0.05, **p < 0.01, ***p < 0.001, and ****p < 0.0001.

## Results

### Transcriptome longitudinal landscape of differentiating NSC

**Figure 1:**
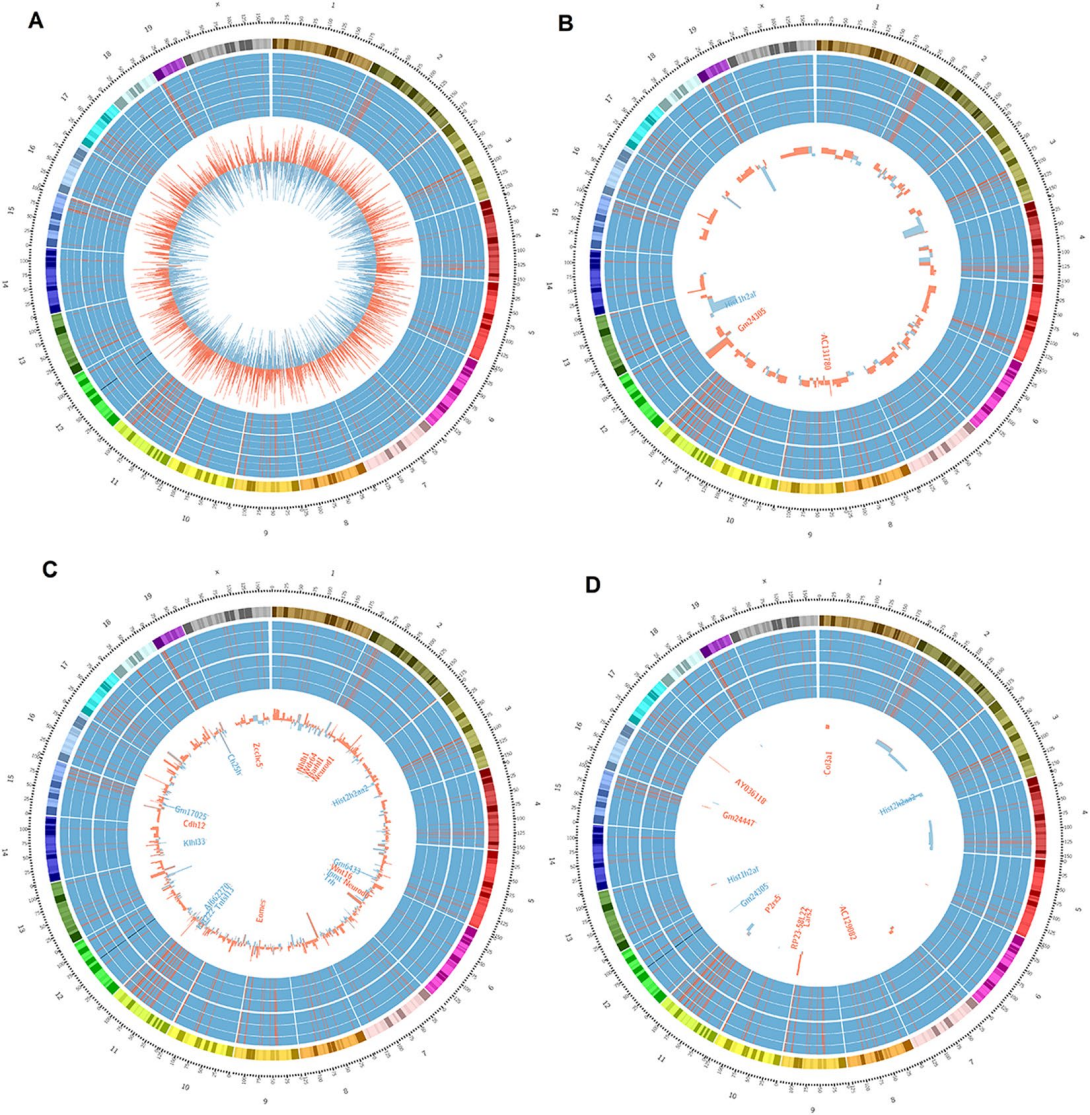
Transcriptome landscapes of different mouse chromosomes in differentiating neural stem cells with or without ethanol treatments. The Circos displays from the outmost ring to the inner rings: chromosome number of mm10 reference genome, cytobands, and heatmaps are shown with three replicates for each condition, and the histogram (shown as the inner most ring). The heatmaps are displayed as RNA-seq reads number per 1 Mb span with high (red) and low (blue). The histogram displays the differential expression between two conditions with up-regulated (red) or down-regulated (blue). (**A**) The transcriptomes of controls. The heatmap displays D0, D2, and D8, each having three replicates, from outer rings to the inner rings. The inner most histogram shows differential gene expression of D8 versus D2. (**B**) The transcriptome of D2. The heatmap displays D2 control (outer) and D2 ethanol binge (inner). The histogram shows the differential gene expression program of D2 control versus D2 ethanol. (**C**) The transcriptome of D8 is shown. The heatmap displays D8 control (outer ring) and D8 continuous ethanol (inner ring). The histogram shows the differential gene expression program of D8 control versus day 8 ethanol. (**D**) The transcriptome of D8. The heatmap displayed D8 control (outer ring) and D8 ethanol withdrawal (inner ring). The histogram shows the differential gene expression program of D8 control versus D8 ethanol withdrawal. The highlighted genes are the 2-fold changes or top 10 genes.

Normal transcriptome profiles of cells at D0, D2, and D8 provided a baseline for studying ethanol effects. By examining RNA-seq reads mapped on gene transcripts with at least normalized 10 reads per gene, we found that 16675, 16550, and 15973 genes were expressed at D0, D2 and D8, respectively. More than 80% of total genes were differentially expressed at different time-points: 13851 genes between D0 and D2, 15441 between D0 and D8, and 12041 between D2 and D8, using a cut-off of adjusted p-value < 0.05. We binned RNA-seq reads within each 1 Mb span among all chromosomes. Hot spots of gene expression were found on chromosome 2, 3, 4, 5, 11, 15, 17, and 29 (Fig. 1A). These hot spots appeared to be active during all time-points from D0-to-D8. A few areas in chromosome 2 and 3 showed lower expression levels at D0, but transcripts increased at D2 and/or D8. On chromosome 14 and 15, a few genes expressed at D0 showed lower expression at D2 or D8. A large number of genes were up-regulated or down-regulated when compared to D2 and D8.

### Longitudinal transitions of biological pathways of differentiating NSC

**Figure 2:**
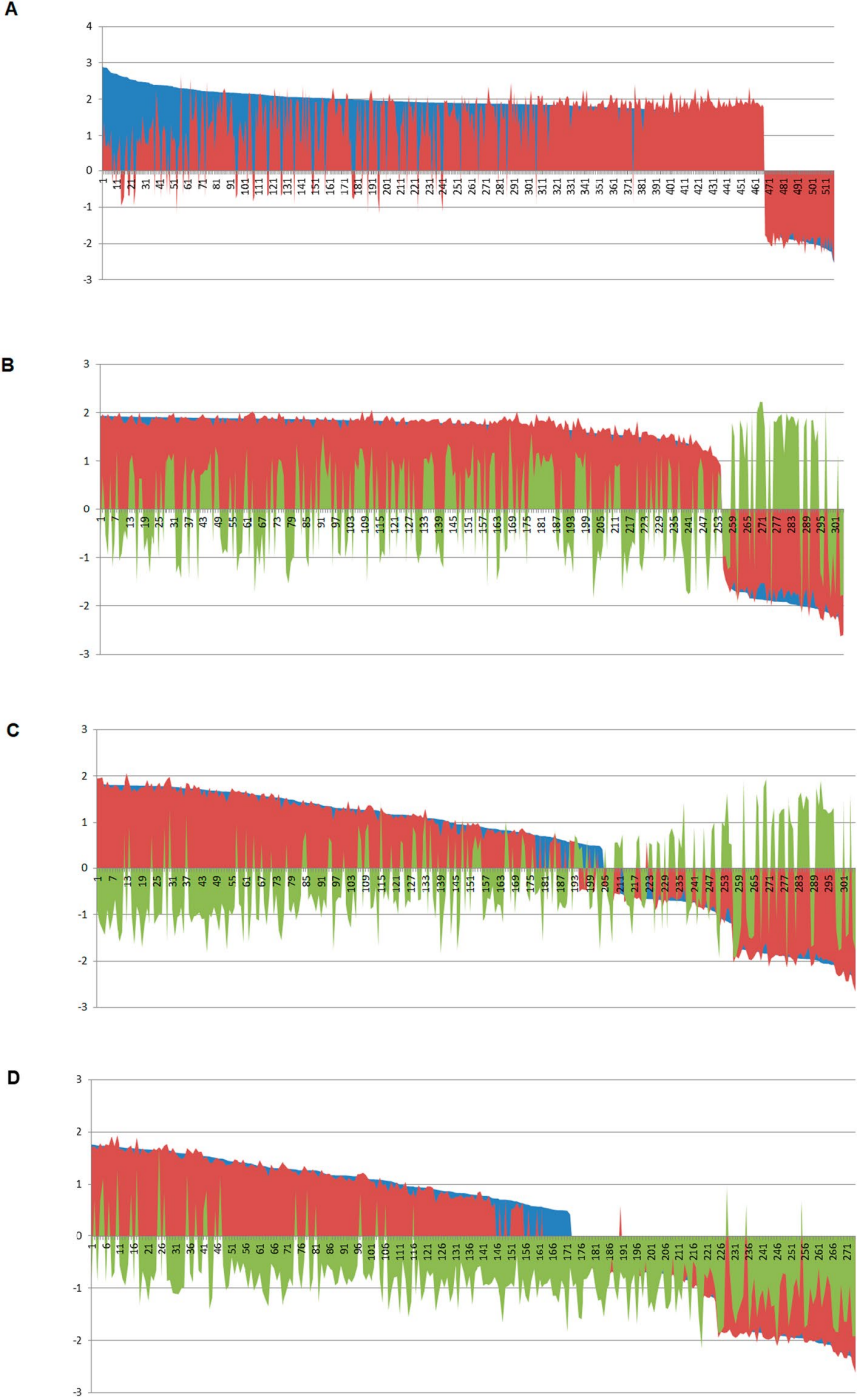
GO (gene ontology) term enrichment analysis. (**A**) Longitudinal transitions of GO-terms of normal control from D2 to D8. The enriched GO-terms of D2 control versus D0 control (blue) was ranked by normalized enrichment score (NES), and then the NES of GO-terms of D8 control versus D0 control (red) were overlaid. (**B**) The effect of GO enrichment profile of D2 ethanol. NES ranking of D2 control versus day 0 control (blue) was overlaid by the NES of D2 ethanol binge versus day 0 (red), and D2 control versus D2 ethanol (green). (**C**) The effect of GO enrichment profile of D8 continuous ethanol. NES ranking of D8 control versus day 0 control (blue) was overlaid by the NES of D8 continuous ethanol versus D0 (red), and D8 control versus D8 ethanol (green). (**D**) The effect of GO enrichment profile of D8  ethanol withdrawal. NES ranking of D8 control versus D0 control (blue) was overlaid by the NES of D8 ethanol withdrawal versus D0 (red), and D8 control versus D8 ethanol withdrawal (green). The Y-axis shows NES and the X-axis is the ranked GO-terms. This shows how the control (red) profile was altered by various ethanol treated profiles (blue, green).

Next, we conducted GO-term enrichment analysis by GSEA at D2 compared to D0. We ranked enriched GO-terms by normalized enrichment score (NES) with false discovery rate (FDR) < 0.05. This rank was set as base-profile of functional gene groups. This profile allowed examining differences with D8. Many highly enriched GO-term groups at D2 became less or negatively enriched (down-regulated compared to D0) at D8 in overall GO-term profile comparisons (Fig. 2A). Few enriched GO groups were higher/up-regulated at D8 compared to D2. All down-regulated GO-term groups at D2 were also down-regulated at D8. The clustering (Supplementary Fig. S2) showed the same pattern of GO function group shift from D2 to D8. Overall, more GO groups shifted down at D8.

We closely examined these up- and down-regulated GO function groups (Supplementary Table S3). Interestingly, at D2, top-ranked enriched GO-terms were cell-cycle related, including, sister chromatid segregation (D2 NES:2.9, D8 NES:0.56), DNA replication (D2 NES: 2.77, D8 NES:0.58), cell cycle phase transition (D2 NES: 2.73, D8 NES: 1.16), nuclear chromosome segregation (D2 NES:2.7, D8 NES:−0.44), DNA packaging complex (D2 NES: 2.64, D8 NES: −0.96), and DNA packaging (D2 NES:2.63, D 8 NES:−0.86).

GO groups at D8 related to responses to ethanol became increasingly enriched compared to D2. For example, positive regulation of response to wounding (D2 NES:1.23, D8 NES:1.97), regulation of reactive oxygen species biosynthetic process (D2 NES:1.19, D8 NES:1.9), positive regulation of response to external stimulus (D2 NES:1.18, D8 NES:1.9), response to IL-6 (D2 NES:1.09, D8 NES:1.86), cellular response to interleukin 6 (D2 NES:0.97, D8 NES:1.85), positive regulation of acute inflammatory response (D2 NES:0.87, D8 NES:1.78).

### Ethanol exposure at D2 impacted differentiation, without largely changing transcriptome landscape

**Figure 3:**
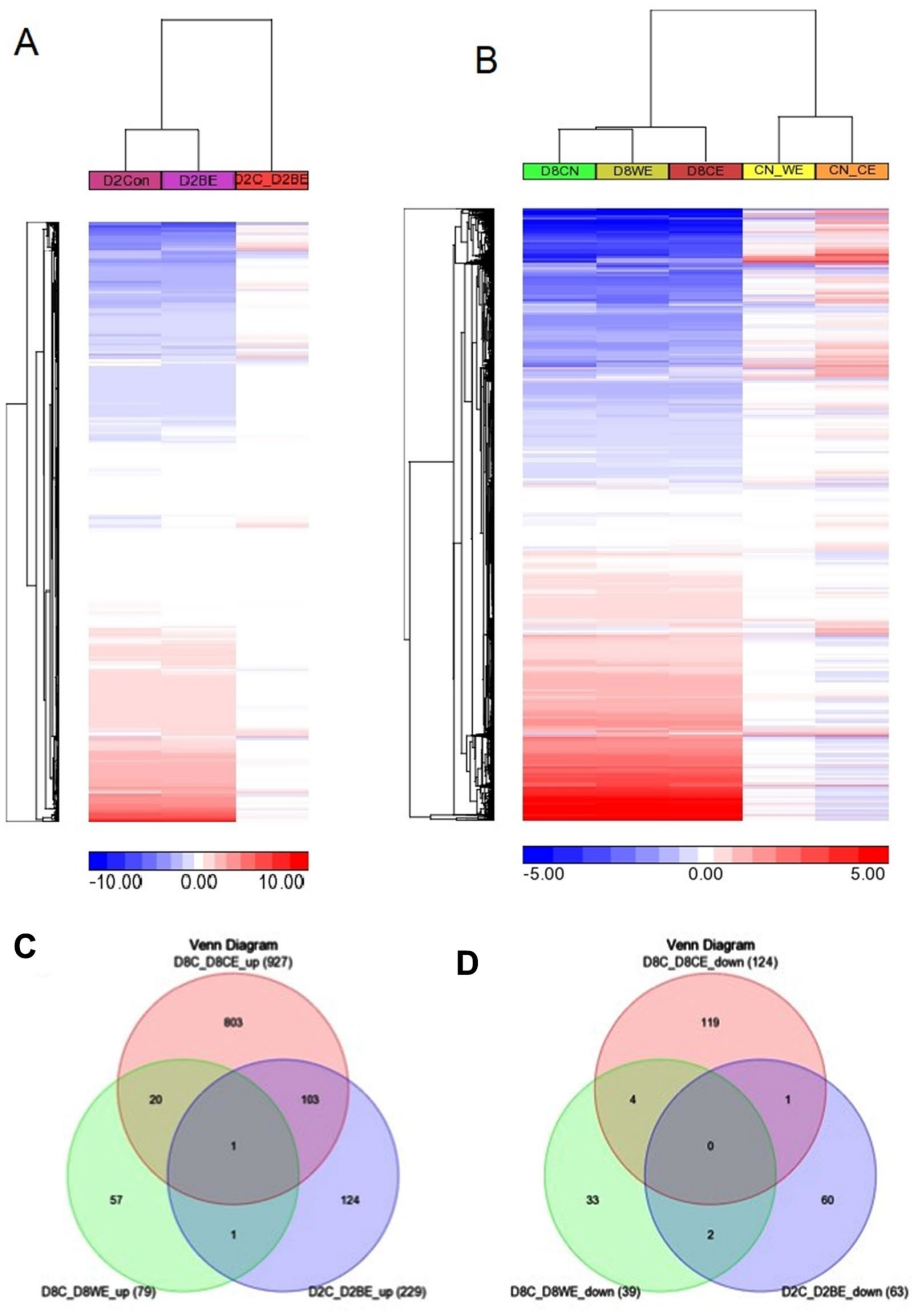
Clustering of differentially expressed genes (DEGs) and their comparison. The log2 fold changes of 20160 mm10 ensemble genes were clustered using 2-dimentsional Euclidean algorithm. Color bar represents the log2 fold change values. (**A**) Three differentially expressed genes (DEGs): D2 control versus D0 control (D2Con); D2 ethanol versus D0 control (D2BE); D2 ethanol versus D2 control (D2C_D2BE). (**B**) Five DEGs: D8 control versus D0 control (D8CN); D8 ethanol withdrawal versus D0 control (D8WE); D8 continuous ethanol versus D0 control (D8CE); D8 ethanol withdrawal versus D8 control (CN_WE); D8 continuous ethanol versus D8 control (CN_CE). The right part of each panel is expression heatmap with color intensity representing differential expression level, for example D8 ethanol withdrawal versus D8 control (CN_WE). (**C**) The overlaps of the up-regulated gene lists in three ethanol treatment conditions. (**D**) The overlaps of the down-regulated gene list in three ethanol treatment conditions. The DEGs were selected using FDR < 0.05 in edgeR analysis.

Examination of global transcriptome at D2 showed a similar pattern with 1 Mb span on all chromosomes between control and D2BE (Fig. 1B). When we compared differential gene expression between D2BE with D2Con, we found 292 genes with FDR < 0.05 including 229 up-regulated and 63 down-regulated genes (Supplementary Table S4 and Fig. S3A). We only applied FDR < 0.05 without using fold change cut-off, which allows inclusion of genes whose little change in expression may have a tremendous impact on biological functions. This is the same day point static comparison (D2Con versus D2BE), which could not give the gene expression trend after ethanol treatment. When we performed clustering of the differential gene expression of D2Con versus D0, and D2BE versus D0, we found that some genes that were (D2Con versus D0) up-regulated were down-regulated by ethanol at D2BE, and some genes that were (D2Con versus D0) down-regulated were up-regulated by ethanol (Fig. 3A). These genes were further highlighted in clustering of D2BE versus D2Con (Fig. 3A, column 3). These genes with direction changes (up- or down-regulated) might be particularly more important than incremental changes in expression. For example, *Nav1* was down-regulated (−1.43 fold change) in D2Con over D0; while in D2BE, it was 1.01-fold change over D0, an opposing direction. As a result of comparing D2BE over D2Con, *Nav1* was up-regulated by 1.34 fold change with FDR of 1.72E-05.

Next, we examined GO-term enrichment of D2Con and D2BE versus D0. We clustered both enrichment gene lists using normalized enrichment score. Although overall profile of GO function groups was retained for normal NSC differentiation, they were not identical and some clustering regions showed different intensity of GO-term NES (Supplementary Fig. S4A). We further ranked GO-terms by NES of D2Con over D0 and found that ethanol at D2BE did not disturb the overall pattern of GO-term ranking. However, when NES of D2 was analyzed for ethanol versus D2Con, few GO functional groups were down-regulated or up-regulated by D2BE (Fig. 2B, Supplementary Table S5). This indicates that at D2 ethanol altered the expression of genes that were associated with NSC differentiation.

### Ethanol exposure at D8 deregulated transcriptome landscape of differentiating NSC

Others and we have shown that a range of ethanol exposure from 22–70 mM does not change NSC differentiation^7,13^. However, RNA-seq results with 1 Mb span plot showed a large area in chromosomes 1, 3, and 4 were decreased in transcription by ethanol, i.e., the high expression with red bars appeared in 3 control samples but disappeared in 3 ethanol treated samples (Fig. 1C). When we further compared defferential gene expression, there were 1051 significantly differentially expressed genes (927 up-regulated, 124 down-regulated, FDR < 0.05) between D8CE and D8CN (Fig. 3B, Supplementary Table S6 and Fig. S3B).

We clustered normal GO-term enrichment of D8Con over D0, and D8CE with D0. Continuous ethanol (D8CE) not only increased or decreased D8Con normal enrichment (same color in Supplementary Fig. S4B), but also disturbed normal GO-term enrichment during the NSC differentiation (GO-term cluster with different colors between D8CE versus D0 and D8Con versus D0). This was further supported by ranking the normal GO-term enrichment and overlaid GO-term enrichment of D8CE (Fig. 2C). The directly compared D8CE over D8Con also showed that the ethanol remarkably impaired the normal GO function group ranking order (GO-terms in green, Fig. 2C, Supplementary Table S7).

### Ethanol withdrawal restored transcriptome landscape without fully reversing altered gene expression of differentiating NSC

The overall transcriptome of D8WE resembled more similarity with D8Con (Fig. 1D) compared to D8CE and D8Con (Fig. 1C). By further clustering the differential gene expression results, we found that withdrawing ethanol (D8WE) restored the transcriptome as it clustered with D8Con, yet it is distinct from D8CE (Fig. 3B). Only 118 genes were significantly expressed (79 up-regulated, 39 down-regulated) between D8WE compared with D8Con (Fig. 1D, Supplementary Table S8 and Fig. S3C), which is much less than the D8CE (Supplementary Table S6).

We ranked GO-term NES of D8Con and observed that high NES GO-terms showed a consistent order between D8WE and D8Con. However, for those GO-terms with lower NES, normal enriched GO-terms were not restored by ethanol withdrawal, but rather decreased (Fig. 2D**)**. In clustering using GO-term NES, D8WE was clustered with D8CE (Supplementary Fig. S4B), which is different from differential gene expression clustering in which, the cluster D8WE was clustered with D8Con (Fig. 3B**)**.

### Genes significantly altered by ethanol in differentiating NSC

We applied FDR < 0.05 for differentially expressed gene cut-off in three gene lists, D2BE versus D2Con (Supplementary Table S4), D8CE versus D8CN (Supplementary Table S6), and D8WE versus D8CN (Supplementary Table S8). Within 229 induced by D2BE, almost half (104) were continuously up-regulated by D8CE **(**Fig. 3C**)**. The majority of 927 up-regulated genes by D8CE were restored to normal after ethanol withdrawal, with 21 common genes. This suggested that with increased time of exposure, the number of affected genes is increased. On the other hand, ethanol withdrawal almost reversed gene expression changes caused by binge ethanol exposure.

Our data set showed that *Nav1* gene was the only one gene that was consistently up-regulated by ethanol under different conditions (Fig. 3C). In contrast, *Serpinf*1 gene was up-regulated at D2BE, but persistently stayed elevated even in D8WE. Interestingly, this gene was not up-regulated in D8CE. D2BE and D8CE shared 104 up-regulated genes (e.g. *Barhl1*, *Cntn2*, *Asxl3*, *Atp1a3*, *Dcc*, *Dcx*, *Dscaml1*, *Gabrg2*, *Rab3c*, *Scn3a* and *Sptbn2*), but only 3 down-regulated genes (*Hist1h2al*, *As3mt*, *Gm25394*) (Fig. 3D). Noticeably, we found more up-regulated genes than down-regulated genes by ethanol. This suggested that ethanol treatment exerted more of a gene induction mode than inhibition.

### Validation of differentially expressed genes by RT-PCR

To validate RNA-seq results, we randomly selected 8–10 genes from D2 and D8 conditions (D2: up-regulated- *Scn3a*, *Gabrg2*, *Kcnq3;* down-regulated- *Rny1*, *Postn*; D8CE: up-regulated- *Synpr*, *Sptbn2*, *Dscam;* down-regulated- *Nfil3*, *Kcns1*). We compared the fold change values from RNA-seq to qRT-PCR data for selected genes normalized to *Gapdh* endogenous control (same RNA samples subjected to RNA-seq). RT-PCR products were sequenced to confirm correct transcript products. Fold change values calculated through two different techniques (RNA-seq and qRT-PCR) were consistent in confirming the validity of the RNA-seq data (Supplementary Fig. S5).

### Verification of gene expressions in another strain model

**Figure 4:**
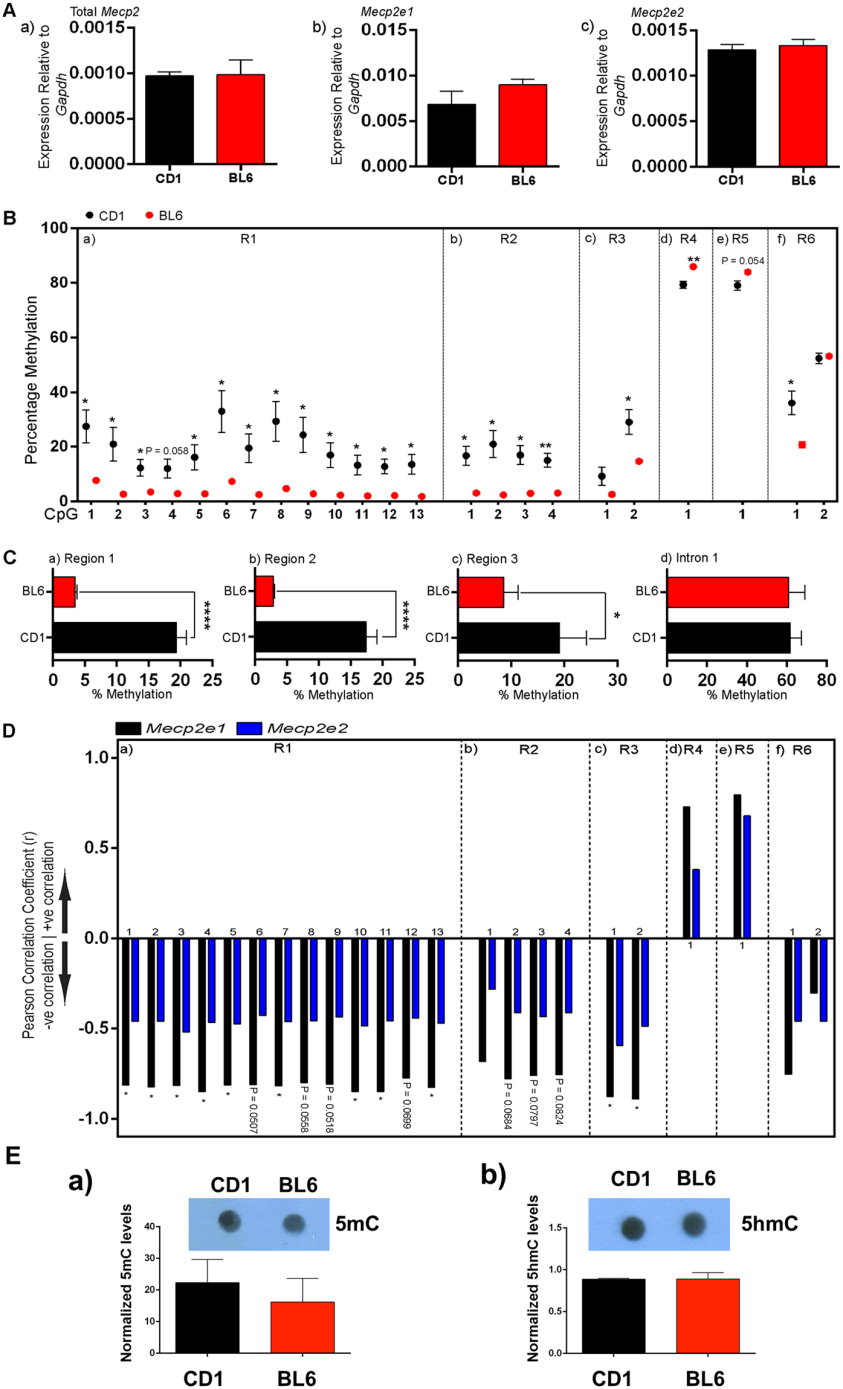
Strain-specific comparison of the *Mecp2* expression and DNA methylation in E14.5 embryonic forebrain of BL6 and CD1 mice. (**A**) Normalized expression of *Mecp2* and its two isoforms (*Mecp2e1* and *Mecp2e2*) compared to *Gapdh* in E14.5 embryonic forebrain. (**B**) DNA methylation at single CpG dinucleotides at the *Mecp2* regulatory regions (R1-to-R6) conducted by Bisulfite pyrosequencing. (**C**) Average DNA methylation from R1-to-R3 from the promoter (individual regions), or R4-R6 from intron 1 (combined three regions). (**D**) Correlation coefficient for DNA methylation at single CpG dinucleotides from R1-to-R6 and *Mecp2e1* and *Mecp2e2* expression. (**E**) DNA dot blot from extracted DNA at E14.5 embryonic forebrain in CD1 and BL6 mice. N = 3 ± SEM. *P < 0.05, **P < 0.01, ***P < 0.001, or ****P < 0.0001.

We did not detect any significant change by ethanol for *Mecp2* gene in BL6 NSC, as in our previous study in CD1 NSC^13^. *Mecp2* expression is controlled by DNA methylation, therefore we studied the possibility of differential *Mecp2* gene expression and DNA methylation at its regulatory elements (R1-to-R3 in the promoter and R4-to-R6 in intron-1) between CD1 and BL6 forebrain at E14.5 (developmental time-point that NSC were isolated). No change for *Mecp2* or *Mecp2* isoforms (*Mecp2e1* and *Mecp2e2*) was detected (Fig. 4A). However, bisulfite pyrosequencing showed significant differences at single CpG-dinucleotides in R1, R2, R3, R4, and R6 (Fig. 4B). This was associated with change in average DNA methylation at R1-to-R3, with significantly higher methylation in CD1 compared to BL6 (Fig. 4C). Correlation of DNA methylation with *Mecp2e1/Mecp2e2* showed significant negative correlation at single dinucleotides in R1 and R3 (Fig. 4D). No global DNA methylation differences (5mC or 5hmC) were detected by DNA dot blot (Fig. 4E). This suggested that DNA methylation differences in R1-R6 are specific, highlighting that *Mecp2* regulation is complex and beyond simple contribution of differential DNA methylation.

**Figure 5:**
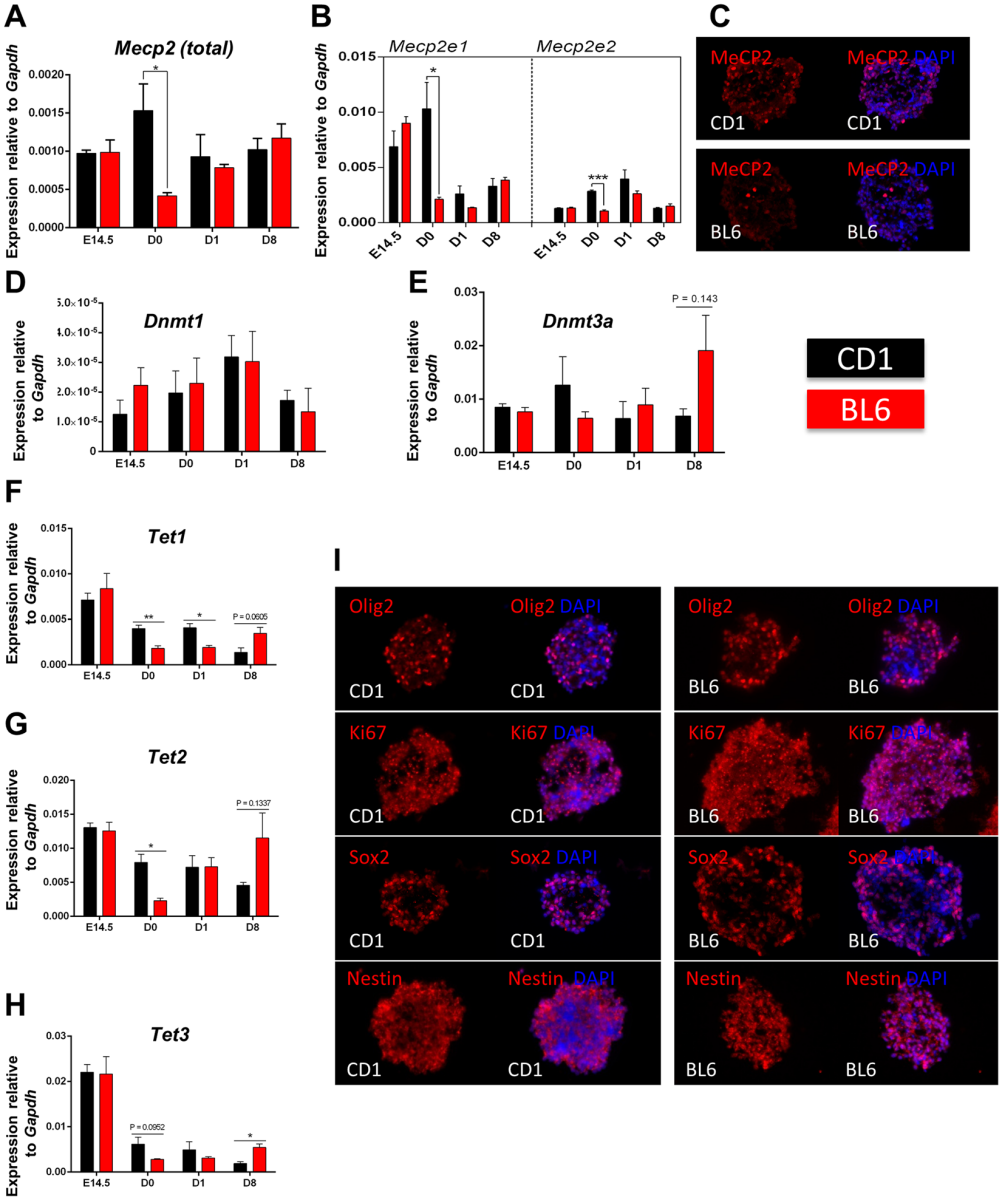
Strain-specific comparison of *Mecp2/*MeCP2, DNA methylation-related genes, and specific markers in neural stem cells of BL6 and CD1 mice. (**A**,**B**) Normalized expression of *Mecp2* and its two isoforms (*Mecp2e1* and *Mecp2e2*) compared to *Gapdh* in E14.5 embryonic forebrain, D0 neural stem cells (neurospheres), D1, and D8 after differentiation in CD1 and BL6 mice. (**C**) MeCP2 expression by IHC in sectioned neurospheres (D0) in CD1 and BL6 mice. (**D**,**H**) Similar to (**A–B**), but for *Dnmt1*, *Dnmt3a*, *Tet1*, *Tet2*, and *Tet3*. (**I**) IHC detection of indicated specific protein markers in CD1 and BL6 sectioned neurospheres (D0). N = 3 ± SEM. *P < 0.05, **P < 0.01, or ***P < 0.001.

Next, we studied whether *Mecp2* transcripts are similar in NSC from CD1 and BL6. No change was detected at E14.5 forebrain, or during differentiation for *Mecp2*, but D0 cells showed higher *Mecp2* in CD1 (Fig. 5A,B). IHC studies of sectioned neurospheres (D0) confirmed higher MeCP2 expressing cells in CD1 neurospheres compared to BL6 (Fig. 5C). Further examination of DNA methylation-related genes (*Dnmt1*, *Dnmt3a*, *Tet1*, *Tet2*, *Tet3*) indicated a trend of higher expression for some of these genes in CD1 neurospheres (Fig. 5D–H). Examination of specific markers for cell proliferation (Ki67), and NSC marker/progenitors (SOX2/NESTIN/OLIG2), did not show significant difference between CD1 and BL6 neurospheres (Fig. 5I). This suggests that at least for *Mecp2* and *Tet* genes, differential strain-specific transcript levels may exist in embryonic NSC.

**Figure 6:**
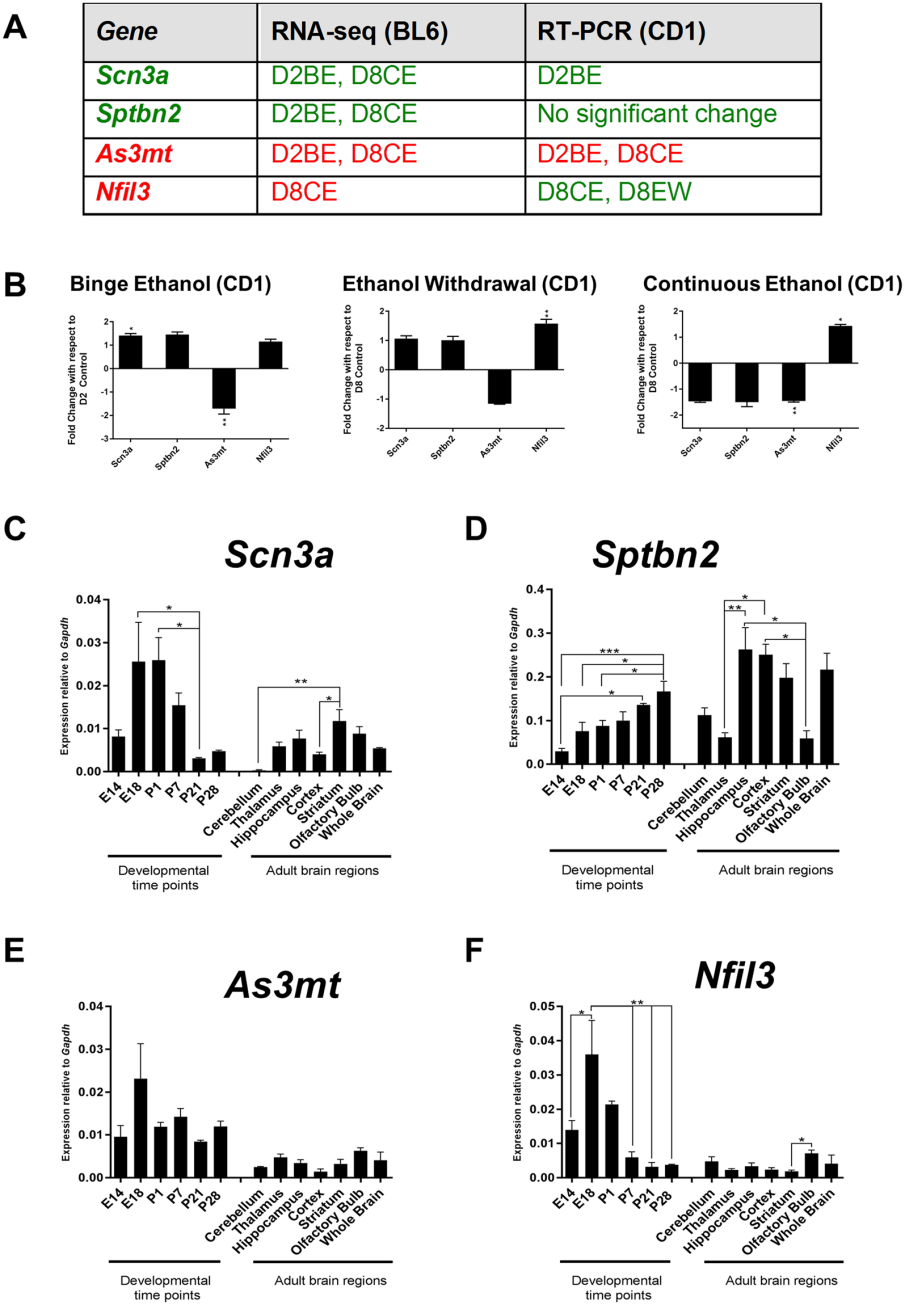
Cross-strain examination of selected genes in BL6 and CD1 NSC exposed to ethanol, along with their developmental and brain region-specific expression. (**A**) Comparison of results from BL6 (RNA-seq) and PCR examination in differentiated NSC exposed to ethanol (D2BE: D2 binge exposure; D8CE: D8 continuous ethanol; D8EW: D8 ethanol withdrawal). (**B**) Relative expression of *Scn3a*, *Sptbn2*, *As3mt*, and *Nfil3* in CD1 differentiated neural stem cells (normalized to *Gapdh*) exposed to different modes of ethanol. (**C**–**F**) Developmental and brain region-specific expression of *Scn3a*, *Sptbn2*, *As3mt*, and *Nfil3* in BL6 mice. Normalized expression is relative to *Gapdh*. N = 3 ± SEM. *P < 0.05, **P < 0.01, or ***P < 0.001.

Furthermore, we selected four more genes (*Scn3a*, *Sptbn2*, *Nfil3*, and *As3mt*) to validate if their expression impacted by ethanol in CD1 strain model. The *Scn3a*, *Sptbn2*, *Nfil3* detected by RNA-seq in BL6 model have first been confirmed by RT-PCR. These three genes are all related to neuronal system. We also chose *As3mt* for its functional role in neurons and possible links to certain phenotypes observed in FASD patients. Cross-examination of these genes showed that *Scn3a* is consistently increased in CD1 and BL6, while *As3mt* is similarly decreased in both strains. However, *Sptbn2* and *Nfil3* were not consistently changed by ethanol in both strains (Fig. 6A,B).

We further investigated the developmental pattern of *Scn3a* and *Sptbn2* that were increased by ethanol, or *As3mt* and *Nfil3*, which showed decreased levels in response to ethanol (RNA-seq). *Scn3a* was expressed in 7 tested brain regions with highest and lowest levels in striatum and cerebellum, respectively, peaking during brain development at E18-to-P1. *Sptbn2* was high in hippocampus, cortex, and striatum, with the highest levels in hippocampus, and low levels in cerebellum, thalamus, and olfactory bulb. During development, *Sptbn2* was increased from E14 till birth and afterwards, with highest levels at P28. *As3mt* expression was consistent in different brain regions and during development. *Nfil3* showed different expression pattern during development, and consistently reduced after birth, with lowest levels at P28 (Fig. 6C–F). Collectively, two of four tested genes which were altered by ethanol in BL6 mice, also showed a similar trend of altered expression by ethanol in CD1 NSC.

### Ethanol onsets abnormal synaptic nervous system via GABA and axonal guidance signalling

In GO-term enrichment analysis using all gene expression data (23368 genes) of D2BE over D2Con, there were many more up-regulated GO-terms than down-regulated GO-terms (Supplementary Table S5). The top enriched GO-terms include synaptic functional groups; indicating synaptic nervous system was abnormally up-regulated by D2.

We then explored the pathways and networks of differentially expressed genes (292 genes) between D2BE and D2Con using IPA. Three of four top networks were related to nervous system development (Supplementary Table S9). Our data indicated that genes associated with nervous system development and functions were abnormally up-regulated (Supplementary Fig. S6A). Network-4 (Supplementary Table S10 and Fig. S7) contains 25 focus gene products including *DCC*, *CACNA1B*, Alpha tubulin that are involved in the canonical pathways of GABA receptor signalling, synaptic long-term depression, axonal guidance and netrin signaling (Supplementary Table S11 and Fig. S8). The GABA receptor signalling appeared to be the most significant signalling pathway that was affected by ethanol binge, which is reported to promote synapse removal and axon shortening in the development of the inhibitory cortical interneurons^30^.

When we examined the potential diseases and functions that could be linked to the 292 genes with differential transcripts, we found that “Development of neuron” and “Behavior” were the top two affected categories with p-values of 5.74E-27 and 1.27E-24, respectively (Supplementary Table S12**)**. This suggests that the binge ethanol first brought about the functional impact on neural system before any profound impact on cellular or other systems.

### Continuous ethanol treatment impaired morphology of embryonic brain cells

We next investigated what diseases and functions were impacted by continuous ethanol treatment. We input into IPA the total 1051 genes that were significantly differentially expressed in D8CE. There were 973 of the 1051 genes, which mapped into IPA database. The top and most significant ones were cancer and cell cycle related (Supplementary Table S13). The “Development of neuron” and “Behavior” were 3.2E-17 and 1.3E-09, respectively, which were far below those non-neural system diseases or functions. This result suggested that continuous ethanol had a profound impact on other cellular functions, including neural functions. After ethanol withdrawal, these “Development of neuron” and “Behavior” were restored, with p-value of 08E-06 and not enriched, respectively (Supplementary Table S14).

**Figure 7:**
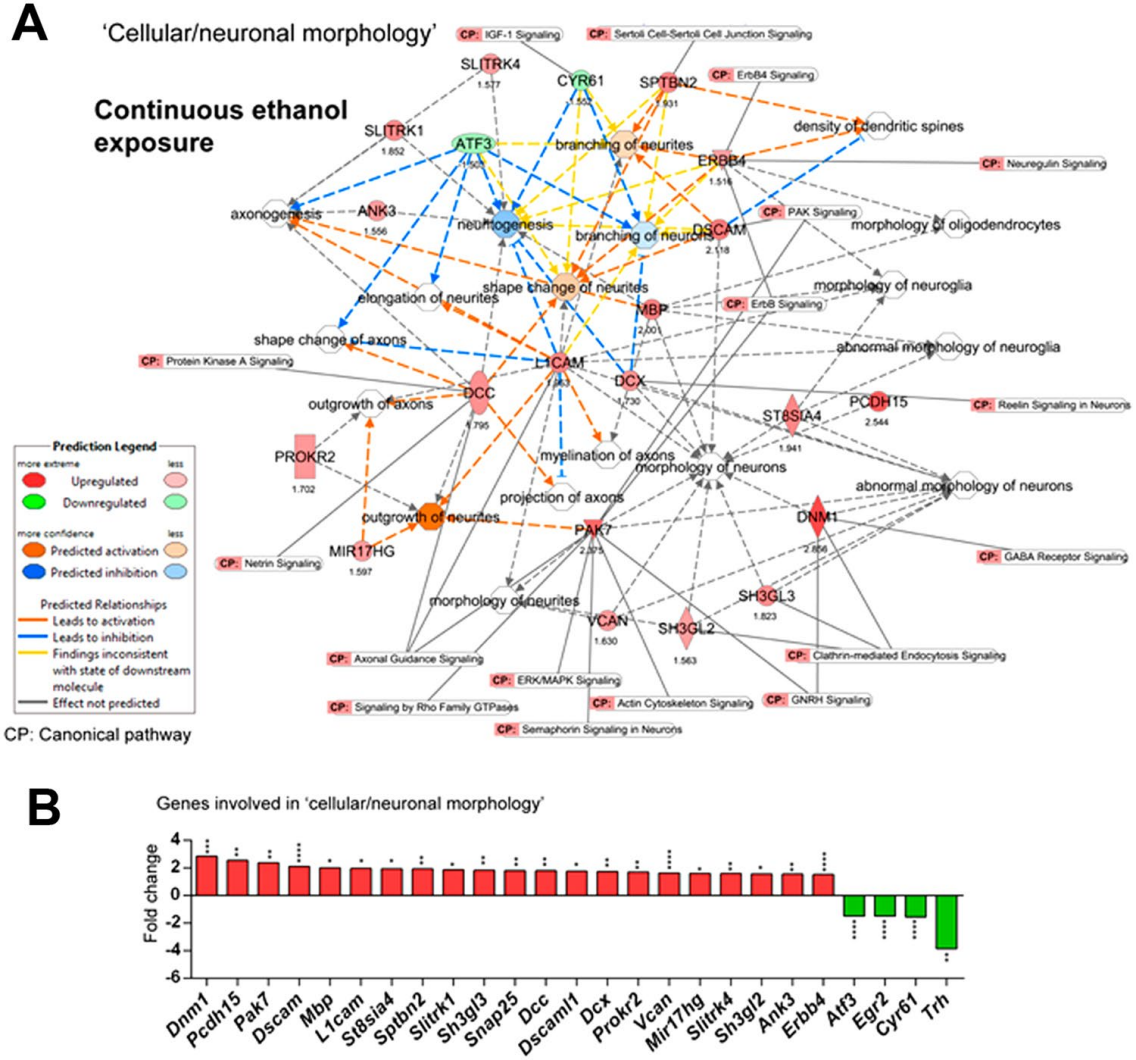
Gene networks and canonical pathways associated with brain/neuronal morphology upon continuous ethanol exposure. (**A**) Overlapping gene networks contributing to different cellular/neuronal morphologies using both significant (FDR < 0.05) and fold changes (>1.5) cut off. The gene expression fold changes within the network are also shown under each gene. “Data were analyzed through the use of IPA (QIAGEN Inc., https://www.qiagenbioinformatics.com/products/ingenuity-pathway-analysis).” (**B**) The fold changes of genes, which are involved in neuronal morphology. Note all genes shown in figure are changed in a statistically significant manner. N = 3. Significant differences from untreated D8 controls are indicated with *P < 0.05, **P < 0.01, ***P < 0.001, or ****P < 0.0001.

Since cognitive and neuro-behavioural abnormalities are reported in patients with FASD, we then studied whether ethanol impacts brain development. We explored the “morphology of brain” in IPA database (Supplementary Fig. S9). In order to explore details of brain morphology and focus, we reanalyzed the DEGs using Cufflinks/Cuffdiff and applied both fold change (>1.5) and FDR 0.05 cut-offs to our gene list and detected several categories of brain structural abnormalities (Fig. 7). Gene expression changes and causal effects analysis in D8CE predicted abnormal morphologies associated with hippocampus, cortex, cerebellum and corpus callosum. Interestingly, induction of *Sptbn2* and *L1cam* by continuous ethanol exposure were linked to induction of the ‘organization of Purkinje cells’ and ‘thickness of the granule cell layer’, in the cerebellum, a part of the brain that is important for different neurological functions^30,30^. Moreover, increased *Dscam* expression was linked to increased thickness of the cerebellar cortex. IPA analysis in both binge and ethanol exposure identified Sptbn2 as a common gene between the two treatment modes, which is associated with increased organization/thickness of granule cell layer of the cerebellum.

Overlaying the canonical pathways with the cellular morphology showed that several major neuronal canonical pathways were connected to genes involved in cellular morphology. Reelin signalling (*Dcx*), calcium signalling (*Atp2b2*), axonal guidance signalling (*Slit1*, *Dcc*), Netrin signalling (*Dcc*) and protein kinase-A signalling (*Dcc*) were among the neuronal signalling pathways that appeared in the analysis. Moreover, *Gfap* was also linked to the Rho family of GTPase signalling.

### Validation of increased neurite branching and disrupted organization of astrocytic cytoskeleton by continuous ethanol exposure *in vitro*

**Figure 8:**
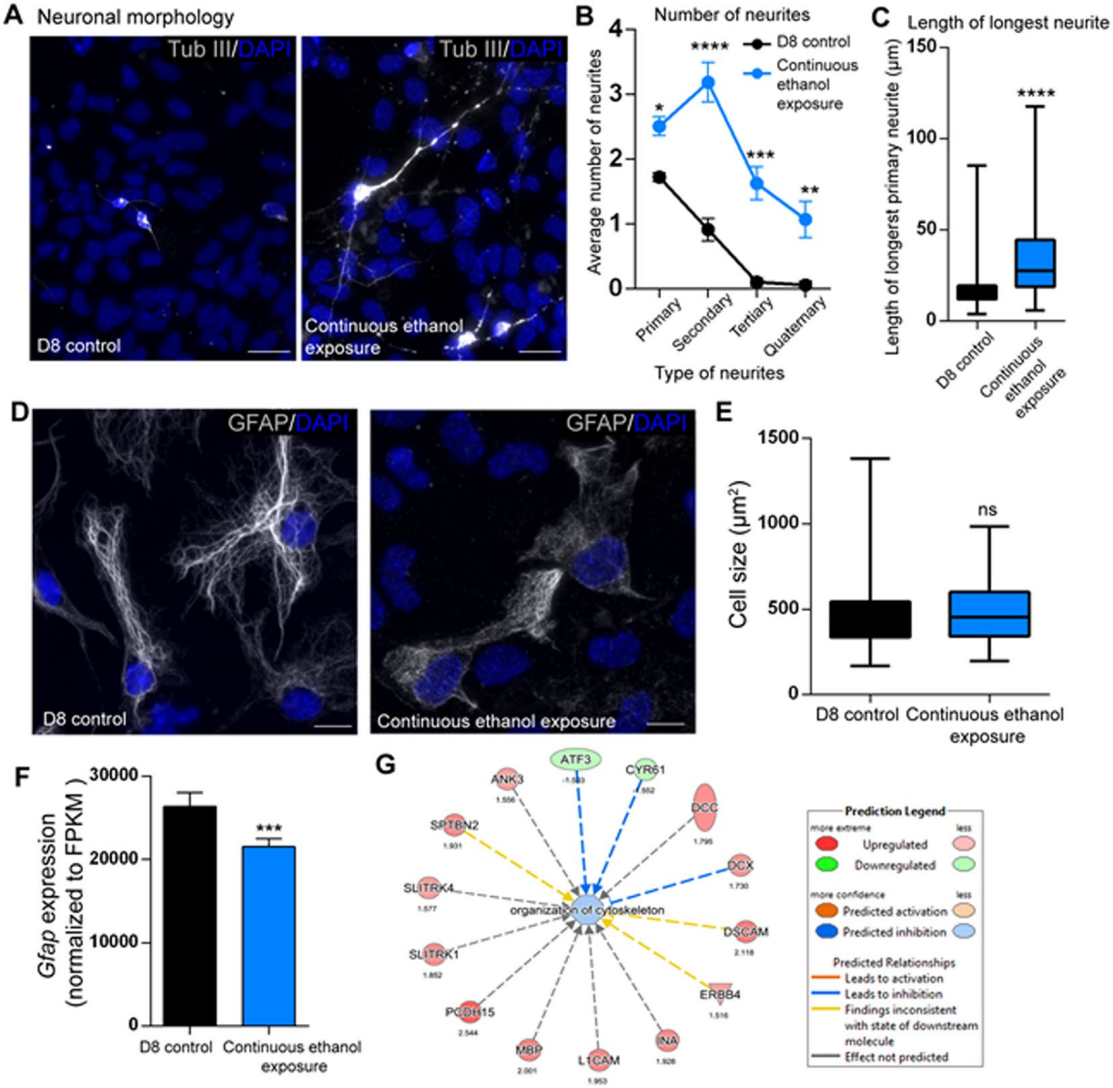
Altered neuronal morphology in continuous ethanol exposure. (**A–C**). Neuronal morphology. **(A**) Comparison of changes in number of neurites in D8 control and continuous ethanol-treated cells. Representative images from D8 control (left panel), continuous ethanol exposure (right panel). Scale bars represent 20 μm. (**B**) Quantification of the number of neurites. From each biological replicate (N = 3), at least 20 TUB III^+^ neuronal cells per were quantified. N = 3 ± SEM. (**C**) Box and whisker plot of the length of the longest neurite in D8 control and continuous ethanol exposure groups. From each biological replicate (N = 3), at least 20 TUB III^+^ neuronal cells per were quantified. N = 3 ± SEM. Significant differences from controls are indicated with P < 0.05, **P < 0.01, ***P < 0.001, or ****P < 0.0001. (**D-E**) Glial morphology. (**D**) Representative images of GFAP^+^ D8 control astrocytes (left), and continuous ethanol exposed astrocytes (right). Scale bars represent 20 μm. Note that no significant change in nuclear size was observed between control and ethanol treatment. (**E**) Comparison of the range of glial cell sizes in (µm^2^) in D8 control and continuous ethanol-treated populations. At least 20 GFAP^+^ cells per biological replicate were quantified under each condition. N = 3 ± SEM. ns: not significant. (**F**) *Gfap* transcript expression values normalized to FPKM (Fragments per kilobase per million reads) in D8 control and continuous ethanol exposure. N = 3 ± SEM. Significant difference from control is indicated with ***P < 0.001. (**G**) Cellular assembly and organization IPA analysis showing the gene networks associated with misregulation of organization of cytoskeleton. “Data were analyzed through the use of IPA (QIAGEN Inc., https://www.qiagenbioinformatics.com/products/ingenuity-pathway-analysis).”

We investigated whether the neuronal morphological changes predicted by gene expression changes were actually represented in neurons. We performed immunocytochemical analysis of β-TubulinIII (Tub-III) in D8CN and D8CE neurons. As predicted by IPA, there was a clear visual difference in morphology between D8CN versus D8CE neurons (Fig. 8A). Quantification of neurite numbers showed significant increase in primary, secondary, tertiary, or quaternary neurites in D8CE neurons (Fig. 8B). We also detected an approximately two-times expansion in the length of the longest neurite (Fig. 8C). While the morphological characteristics on neurons were impacted, the number of TUB^+^ neurons between D8CN and D8CE remained unchanged.

In IPA, morphological properties of differentiated astrocytes were predicted to be through *Gfap*, which in turn was predicted to impact axonal outgrowth of differentiated neurons. Therefore, we examined GFAP^+^ glial cells (astrocytes) for morphological changes at D8CE. Immunofluorescent staining of GFAP^+^ astrocytes showed that in contrast to the bright and well-organized astrocytes in controls, D8CE astrocytes had compromised and disorganized GFAP staining (grainy-like appearance) (Fig. 8D). Quantification of cell surface area (size) did not show any significant size differences between D8CN versus D8CE GFAP^+^ astrocytes (Fig. 8E). The number of GFAP^+^ astrocytes was unchanged between D8CN and D8CE. Moreover, the nuclear size of astrocytes was unaffected by continuous ethanol treatment. As the immunocytochemistry experiments showed reduced intensity of GFAP signals, we further looked at *Gfap* transcripts in D8CE in our RNA-seq datasets. We observed that D8CE caused slight, but significantly reduced *Gfap* expression (−1.23-fold; P = 0.00018) (Fig. 8F). The disorganized GFAP staining of astrocytes could also be caused by disruption of the astrocytes’ cytoskeleton. Interestingly, IPA analysis of cellular assembly and organization at D8CE predicted deregulated organization of cytoskeleton and potential inhibition of cytoskeleton organization **(**Fig. 8G). Based on the predictions, induced expression of *Dcx* and reduced expression of *Atf3* and *Cyr61* could be directly associated with inhibition of cytoskeleton organization. Collectively, our data suggest that continuous ethanol causes reduced *Gfap* expression and organization of astrocytic cytoskeleton, without affecting astrocyte sizes.

## Discussion

Using an established model of NSC differentiation^13^, we studied the global impact of ethanol on cellular gene expression through RNA-seq studies. This cellular model consisted of differentiated NSC, representing diversified population of nervous system cells (i.e. oligodendrocytes, neurons, astrocytes)^14,33^. Several different *in vivo* and *in vitro* models have been used to study prenatal ethanol exposure and alteration in gene expression. However, the common impact may include change in DNA methylation and altered level of genes connected to neurogenesis. Yuan *et al*. reported that altered *Bcl-2* expression in response to ethanol is associated with change in histone modifications at its promoter, which can be recovered by Sulforaphane treatment^34^. Additionally, *in vivo* embryonic alcohol exposure with *ex vivo* culture conditions has identified genes with roles in cellular growth and proliferation^35^. Other studies have reported differential expression of transcription factors and genes involved with pluripotency, cell lineage markers, cellular proliferation, and cell signalling molecules^36,37^. We previously reported altered expression of *Mecp2* gene (in CD1 NSC) by ethanol^13^, which was not observed here (in BL6 NSC). Our cross-examination of gene expression in these strains indicated that in addition to *Mecp2*, some other genes have strain-specific expression and response to ethanol. This highlights that discrepancy in genes affected by ethanol exposure reported by independent groups, might be due in part to differences in methodology and/or model systems. By whole genome RNA-seq rather than studying specific targets, our results are not limited to already established ethanol gene targets. This allowed us to identify potential novel markers that might be associated with embryonic ethanol exposure in differentiating brain cells.

We performed whole genome RNA-seq on three ethanol conditions: D2BE, D8CE, and D8WE. We found synaptic functions were significantly impacted by ethanol predicted by IPA to be *via* GABA receptor signalling. These suggested the possibility of D8CE ultimately leading towards change in neuronal morphology, neurite branching, dendritic outgrowth, axonogenesis, and axonal outgrowth. Based on gene interactions, key gene expression changes towards increased neurite branching and outgrowth, included *Sptbn2*, *Slit1*, and *Dcc*, up-regulated by D2BE. However, two genes that were predicted to influence axonal outgrowth (*Gfap* and *Dcc*) had opposing effects, which might lead to moderate or cumulative effects on axonal length. Among the identified genes, *Sptbn2* and *Dcc* stood out from gene networks, to be associated with increased neurite branching in D2BE and D8CE. Increased expression of *Sptbn2* and *Dcc* by D2BE and D8CE was also associated with increased neurite branching. Interestingly, *Sptbn2* was identified in association with ethanol exposure-induced changes in cerebellar thickness and organization. The identified increased number of primary neurites suggested neurite outgrowth induced by D8CE, in agreement with IPA. *Sptbn2* was predicted to contribute to cerebellar structural changes. In the literature, there is no reported link between *Sptbn2* and alcohol exposure, and our study suggests a potential role for *Sptbn2* in alcohol-induced neuronal and brain morphological abnormalities, specifically in cerebellum. Accordingly, *Sptbn2* is reported in connection with cerebellar ataxia^38^. Both D2BE and D8CE shared specific signalling canonical pathways, including Reelin signalling (*Dcx*), Netrin signalling (*Dcc*), and protein kinase A signalling (*Dcc*). Some of shared signalling pathways were connected through different molecules. For instance, axonal guidance signalling in D2BE was linked through *Slit1* and *Dcc*, but through *Pak7*, *Dcc* and *L1cam* in D8CE.

Here, we found that D2BE mainly exerted gene up-regulation. Although D2BE did not disturb overall GO-term profile during differentiation, at D2, few GO functional groups were down-regulated or up-regulated by D2BE. D2BE also affected the neural system “Development of neuron” and “Behavior”. The overall damage to fetus may also depend on risk elements involving total alcohol amounts, period of exposure, and quantity of consumed alcohol each time, and other environmental factors. Our data suggest that by increased duration of ethanol exposure from D2BE to D8CE, number of altered genes was increased, bringing about a profound cellular impact, including neural functions. Although D8WE restored most of gene expression changes in differentiating NSC, some down-regulated genes, particularly deregulation of epigenetics genes such as *Hist1h2al*, *Hist2h2aa2* were not rescued. Finally, altered gene expression not only depends on protein translation but also on protein turnover. Thus, minimal gene/transcript expression changes observed in D8WE may not necessarily indicate reversal of changes, which is a shortcoming of transcript analysis alone.

Our immunofluorescence experiments confirm that ethanol exposure increased neurite branching/outgrowth, and axonogenesis and provided evidence for impaired glial cytoskeleton organization, in agreement with a previous report^13^. Collectively, our results provide insights on potential of these cellular morphological changes as contributing factor(s) in increased size of cerebellum and cerebellar cortex, reported in FASD patients^3^.

We found that *Nav1* gene as a single gene that was consistently up-regulated by ethanol (Fig. 3C). *Nav1* belongs to the neuron navigator family, mainly expressed in nervous system^39^. The encoded protein has characteristic of ATPases with a wide range of cellular functions. *Nav1* is similar to *Unc-53*, which is involved in axon guidance in *Caenorhabditis Elegans*, and is suggested to be important for neuronal development^39^. *Barhl1* is reported to be important for preservation of neurons in zonal layer of superior colliculus, part of brain stem controlling behaviour^40^. *Cntn2/Tag1* is reported to play important roles in the axonal connections of developing nervous system _ENREF_31^41^. *Barhl1* and *Cntn2* were significantly up-regulated by D2BE, with fold change of 1.42 (FDR 1.01E-05) and 1.34 (FDR 8.86E-06), respectively (Supplementary Table S5).

In summary, this study focused on molecular gene expression landscape changes with ethanol, aiming to delineate the functional and cellular impacts. Our study identified potentially important genes and signalling pathways that shed light into FASD pathobiology. We validated a few resulted genes by RT-PCR and in another strain. In particular, we showed gene-specific DNA methylation at the *Mecp2* regulatory elements in these two mouse strains, which may contribute to significant differences for basal expression of *Mecp2* and other DNA methylation-related genes between CD1 and BL6 mice. We attempted to validate one of our hypotheses that astrocytic cytoskeleton is disrupted by ethanol. Future *in vivo* studies in animal FASD models and human patients would strengthen our results.

## Electronic supplementary material

Supplementary Figures S1 to S9, and Supplementary Table S1-S2

Data set 3–14 on separate sheets

## Data Availability

Additional information on the protocols, data, and material are available to the readers upon request.
