# Supplementary material for: Genome-Wide Transcriptome Landscape of Embryonic Brain-Derived Neural Stem Cells Exposed to Alcohol with Strain-Specific Cross-Examination in BL6 and CD1 Mice
Source: Sci Rep. 2019 Jan 18;9:206. doi: 10.1038/s41598-018-36059-y (PMC6338767; doi:10.1038/s41598-018-36059-y)
Supplement: Supplementary file 1 — Supplementary Figures S1 to S9, and Supplementary Table S1-S2 [file 41598_2018_36059_MOESM1_ESM.pdf]

Genome-Wide Transcriptome Landscape of Embryonic Brain-Derived Neural Stem Cells Exposed to Alcohol with Strain-Specific Cross-Examination in BL6 and CD1 Mice

Wayne Xu [1,2], Vichithra R.B. Liyanage[1,3], Aaron MacAulay[1,3], Romina D. Levy[1,3], Kyle Curtis[1,3], Carl O. Olson[1,3], Robby M. Zachariah[1,3], Shayan Amiri[1,3], Marjorie Buist[1,3], Geoffrey G. Hicks[1,3], James R. Davie[1], Mojgan Rastegar[1,3,*]

[1]Department of Biochemistry and Medical Genetics, Max Rady College of Medicine, Rady Faculty of Health Sciences, University of Manitoba, Winnipeg, Manitoba, Canada; [2]Research Institute of Oncology and Hematology, CancerCare Manitoba, Winnipeg, Manitoba, Canada; [3]Regenerative Medicine Program, Max Rady College of Medicine, Rady Faculty of Health Sciences, University of Manitoba, Winnipeg, Manitoba, Canada
*mojgan.rastegar@umanitoba.ca

SUPLEMENTARY INFORMATION

Page 2-10: Figures S1 to S9
Page 11-12: Table S1 and S2

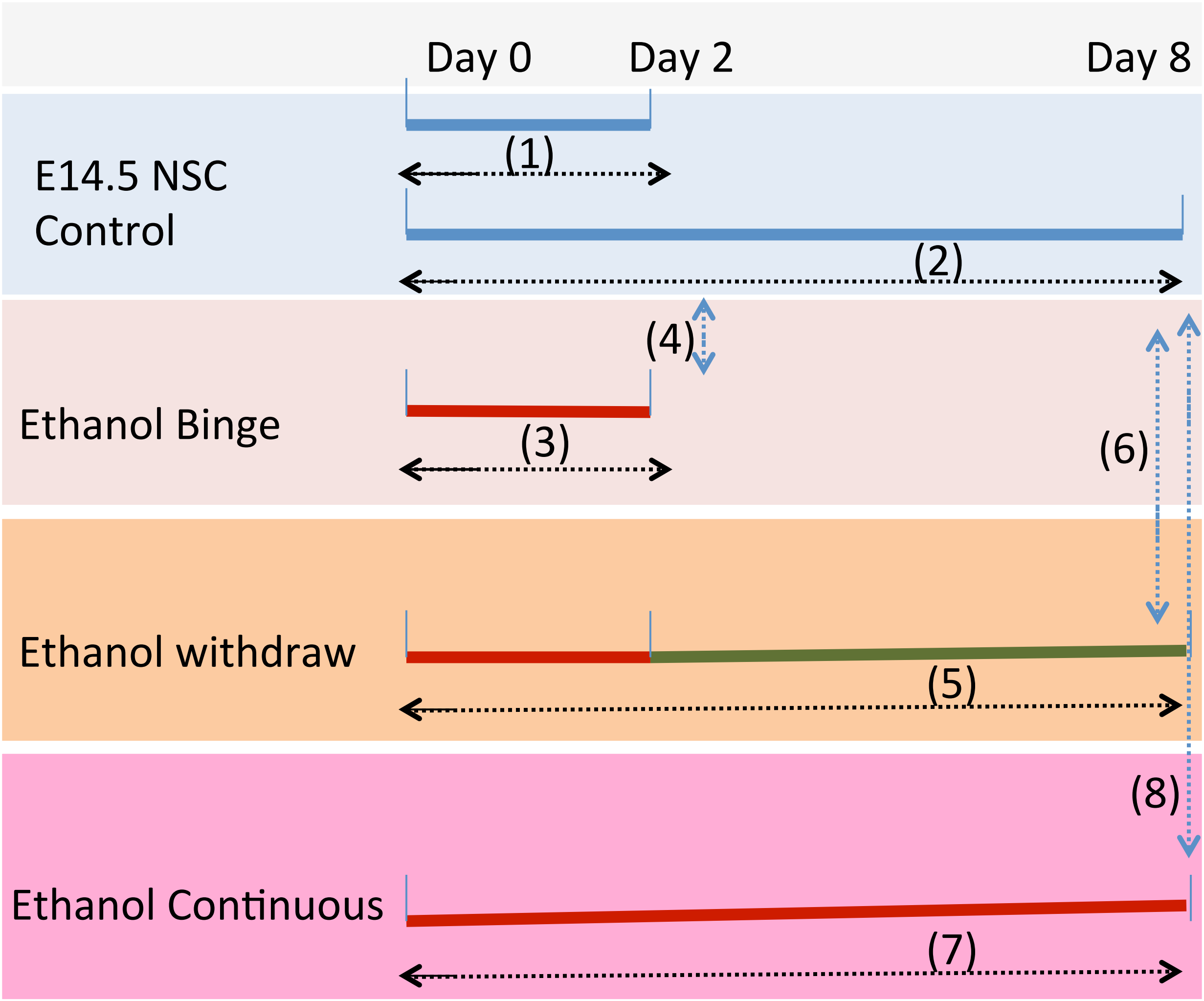

Supplementary Figure S1. Experimental and analytic design. RNA samples were extracted at different days (D0, D2, D8) of different types of ethanol treatments. The differential genes and pathways were analyzed by eight different comparisons as indicated in the Figure by dotted arrow-lines. E: embryonic day; NSC: neural stem cells.

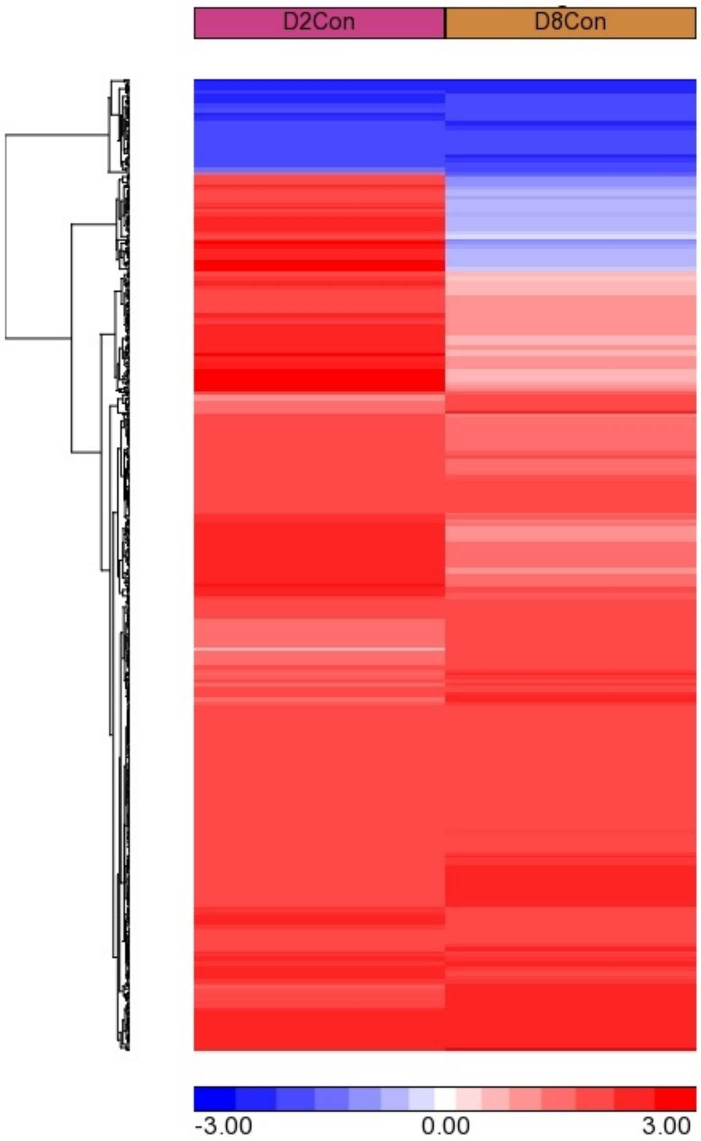

Supplementary Figure S2. Normal control GO function group shift from day (D) 2 to D8. The GO-terms were clustered using the normalized enrichment scores (NES).

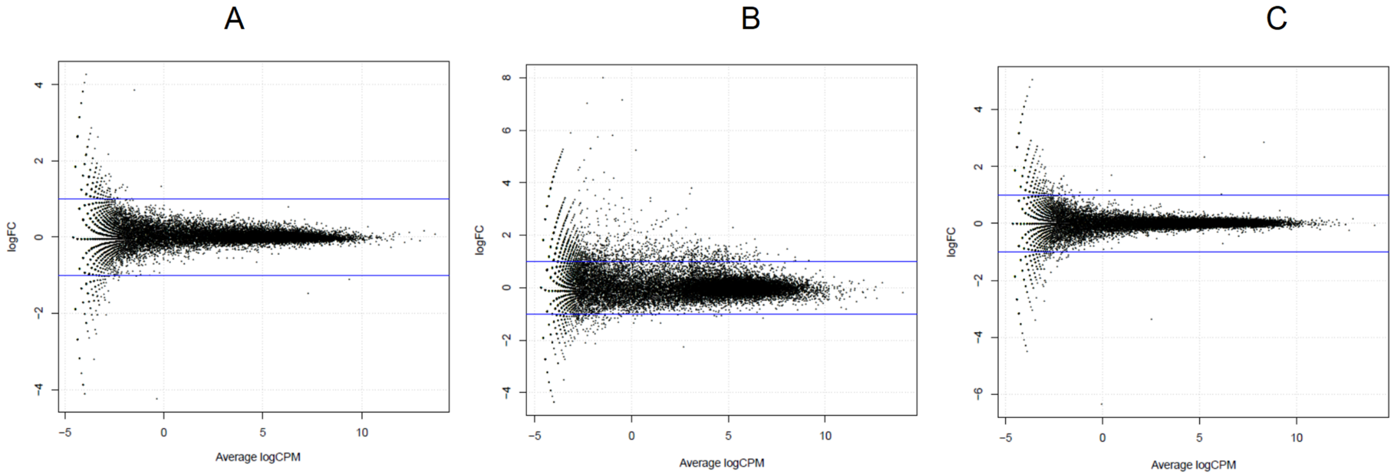

Supplementary Figure S3. The overall distribution of fold change (logFC) versus the gene expression level (logCPM). (**A**) Gene expression of day (D)2 ethanol binge over D2 control. (**B**) Gene expression of D8 continuous ethanol over D8 control. (**C**) Gene expression of D8 ethanol withdrawal over D8 control. Blue lines indicate the 2 fold changes.

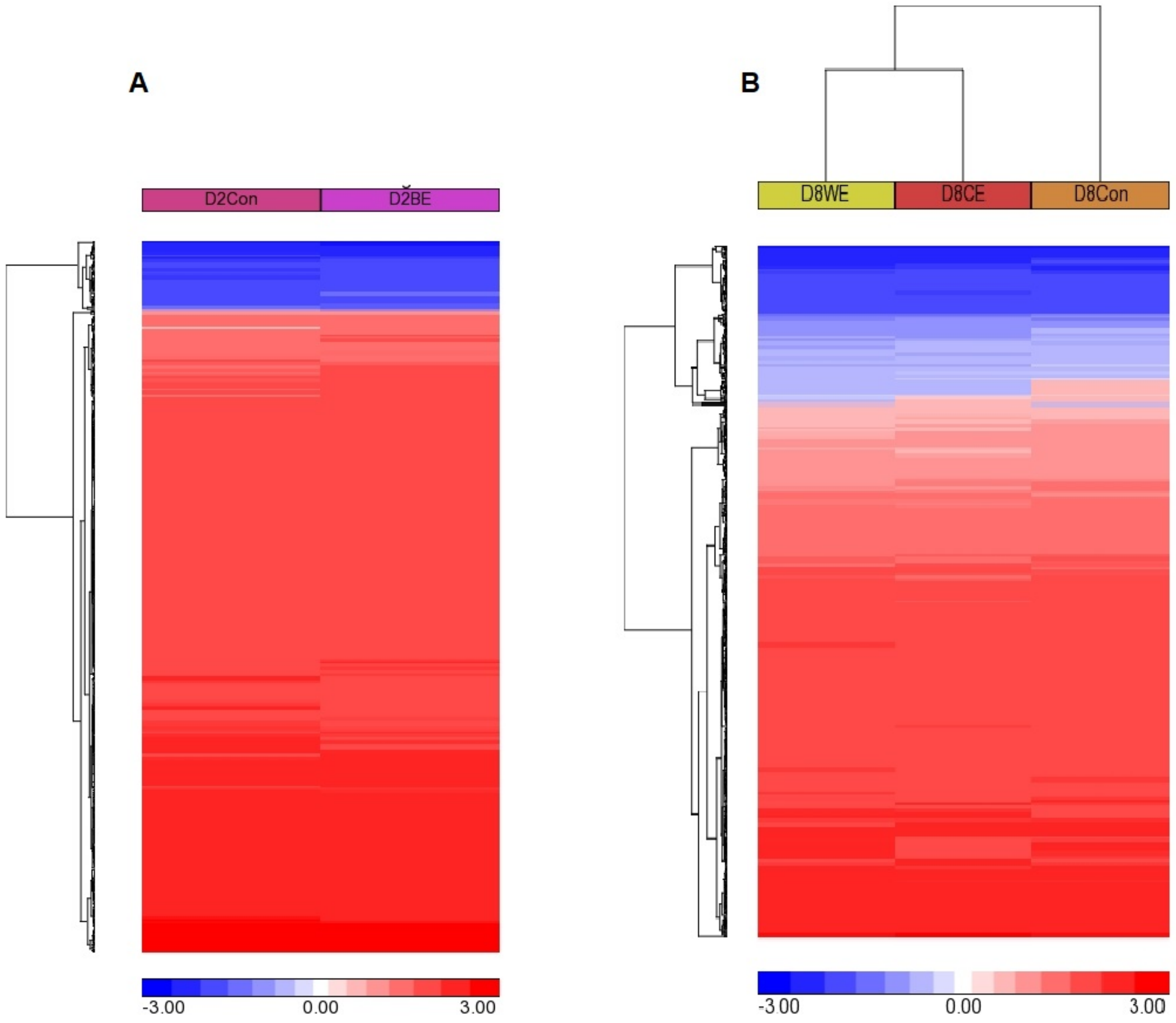

Supplementary Figure S4. Clustering of enriched GO-terms. Normalized enrichment scores (NES) of 517 GO-terms were clustered using 2-dimensional Euclidean algorithm. (**A**) D2Con: enriched GO-terms in day (D)2 control versus D0 control. D2BE: enriched GO-terms in D2 ethanol versus D0 control. (**B**) D8WE: enriched GO-terms in D8 ethanol withdrawal versus D0 control. D8CE: enriched GO-terms in D8 continuous ethanol versus D0 control. D8 Control: enriched GO-terms in D8 control versus D0 control. Color bar represents NES scores. This information refer to all ethanol groups versus D0 control with 2-dimension clustering and showed an overall difference.

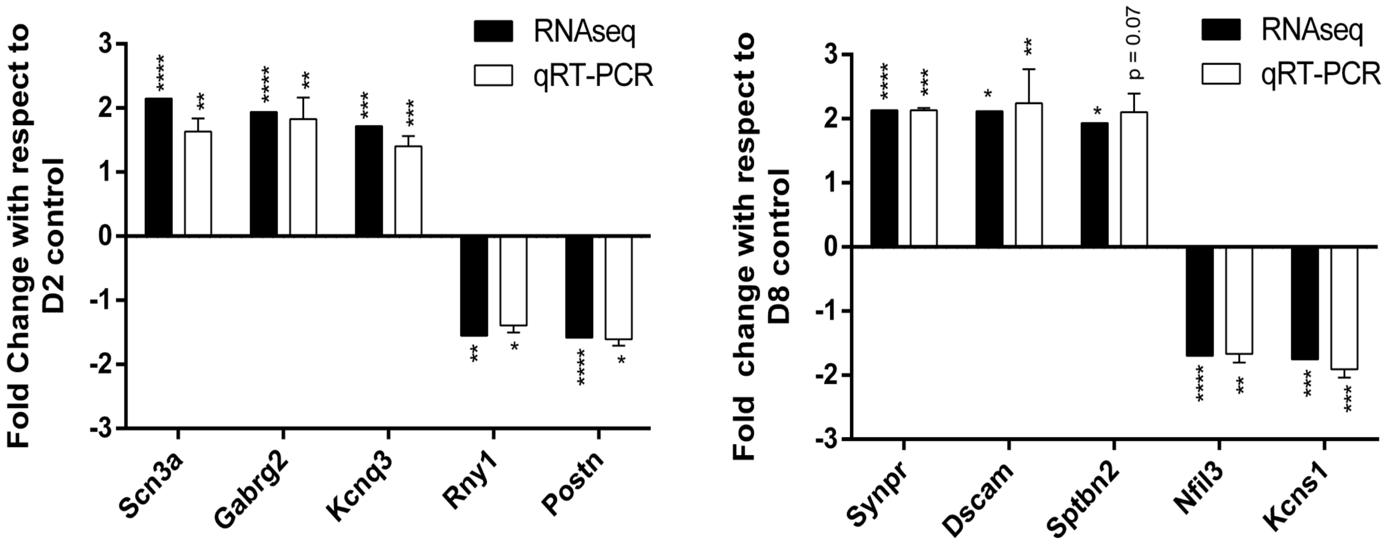

Supplementary Figure S5. Validation RNA-seq data for binge and continuous ethanol exposure using qRT-PCR. (**A**-**B**) The fold change values obtained from RNA-seq analysis was compared to that calculated for data obtained from qRT-PCR experiments. Fold change was calculated with respect to D2 control for Binge ethanol exposure and D8 control for continuous ethanol exposure. For qRT-PCR, the gene expression was normalized to *Gapdh* endogenous control. Quantitative RT-PCR experiments were performed with the same three sets of RNA samples sent for RNA-seq. N=3 ± SEM. Significant differences from untreated controls are indicated with P<0.05* or P<0.01**, P<0.001***, or P<0.0001****.

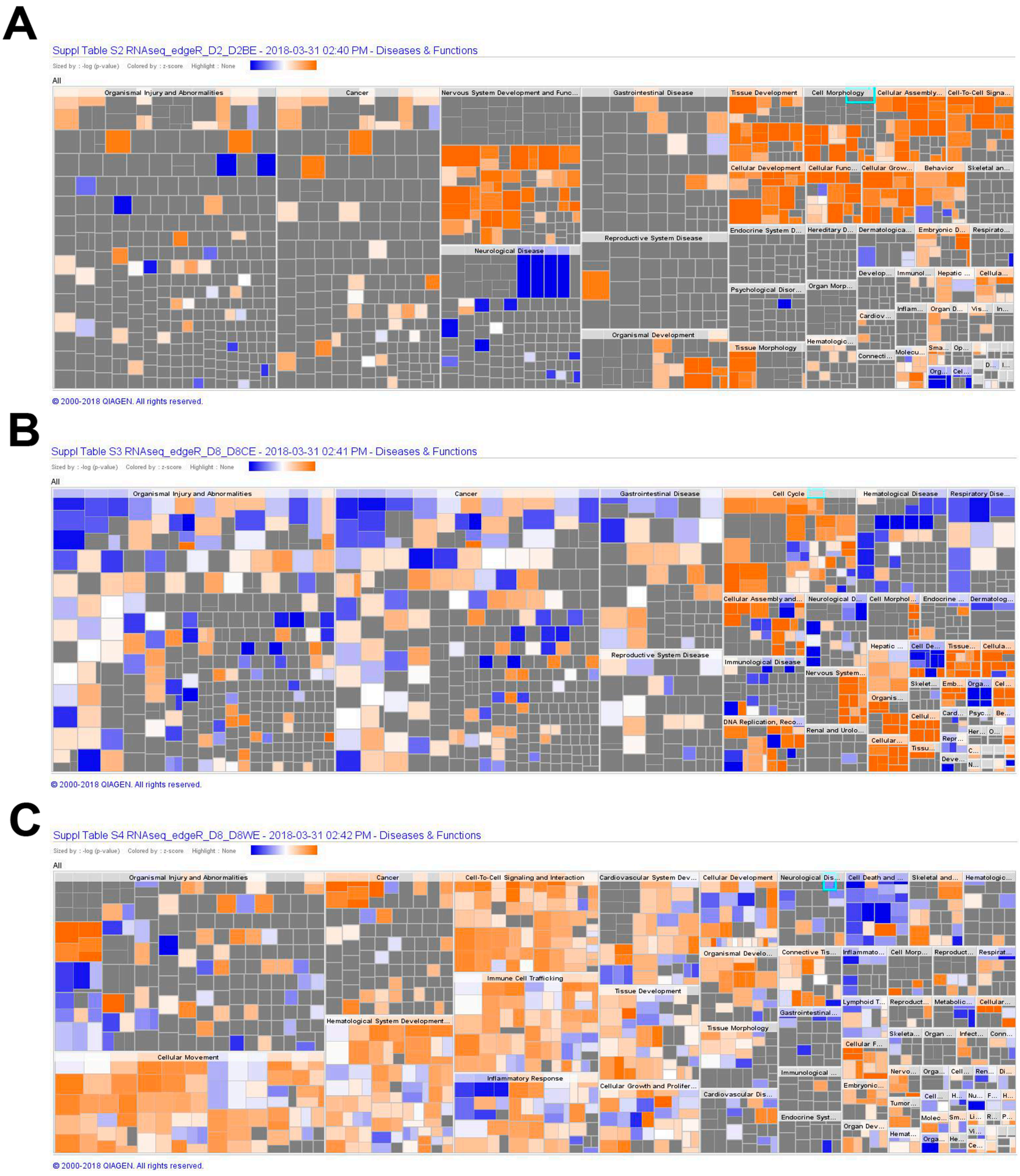

Supplementary Figure S6. Overall the DEGs associated Functions and Diseases in IPA analysis. The names of the Disease or Function groups were labelled. The enrichment z-score calculated from the differential gene list represents the up-regulated (orange) or down-regulated (blue). (**A**) Day (D)2 ethanol associated. (**B**) D8 continuous ethanol associated. (**C**) D8 ethanol withdrawal associated.

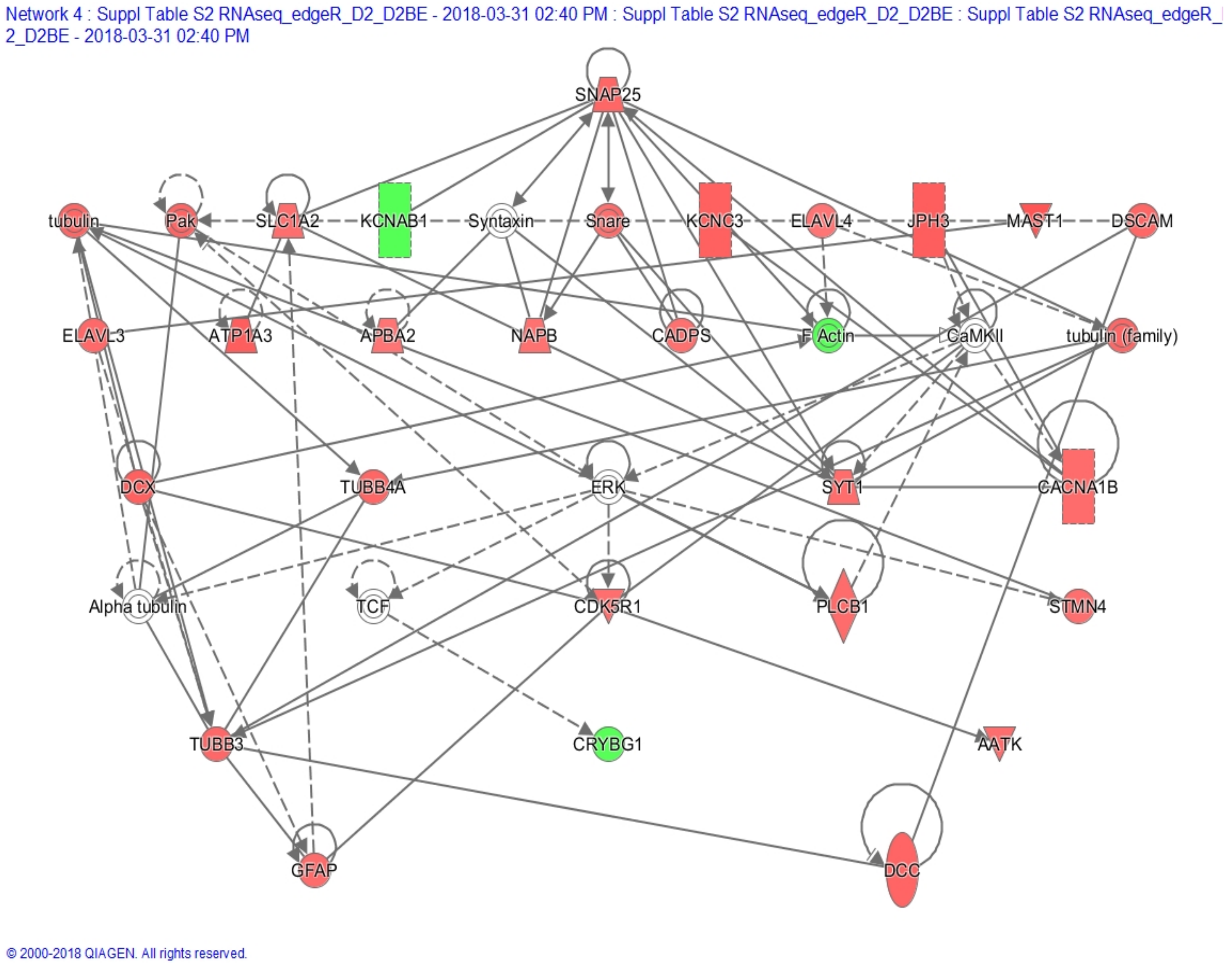

© 2000-2018 QIAGEN. All rights reserved.

Supplementary Figure S7. Network-4 of 2 days (D) ethanol detected by IPA. We found 292 genes that were differentially expressed between D2 ethanol versus D2 control with FDR < 0.05 were inputted into IPA. The network-4 was associated with Nervous System Development and Function, Neurological Disease, Behaviour, with enrichment score of 44. Genes in red represents up-regulated, and genes in green were down-regulated. “Data were analyzed through the use of IPA (QIAGEN Inc., https://www.qiagenbioinformatics.com/products/ingenuity- pathway-analysis).”

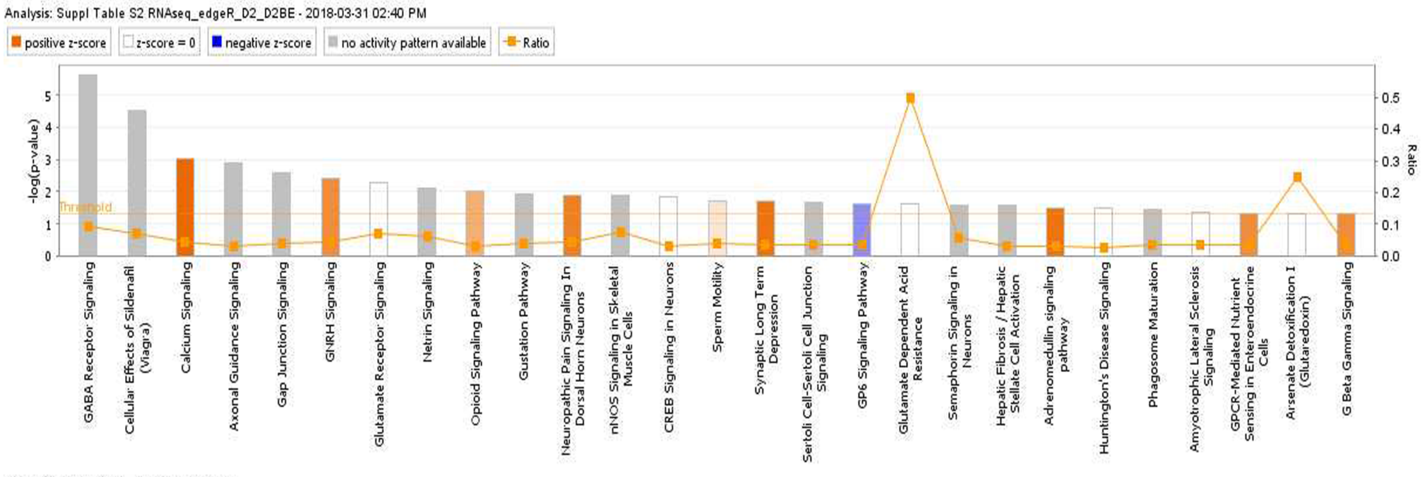

© 2000-2018 QIAGEN. All rights reserved.

Supplementary Figure S8. Canonical pathways associated with day (D)2 ethanol binge. The GABA Receptor signaling was the top pathway. “Data were analyzed through the use of IPA (QIAGEN Inc., https://www.qiagenbioinformatics.com/ products/ingenuity- pathway-analysis).”

Morphology of brain 2

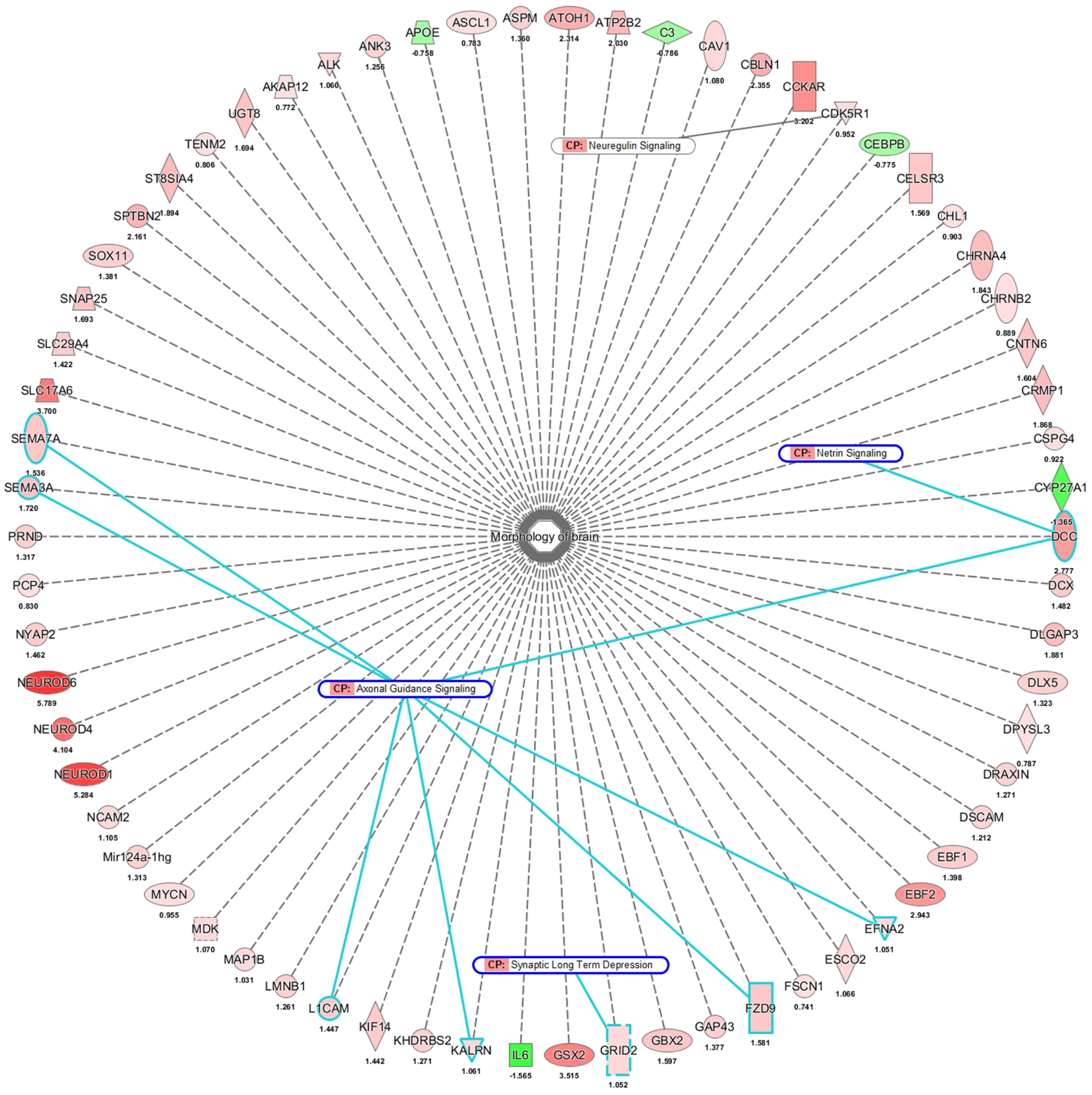

© 2000-2018 QIAGEN. All rights reserved.

Supplementary Figure S9. Gene networks associated with brain/neuronal morphology following long-term ethanol exposure. In function and disease analysis by IPA using all 1051 significant DEGs (FDR<0.05), the Morphology of Brain was significantly (p-value <1.6E-08) associated with the continuous ethanol exposure. Canonical pathways (CP) that are overlapping with the gene networks are also shown. “Data were analyzed through the use of IPA (QIAGEN Inc., https:// www.qiagenbioinformatics.com/products/ingenuity- pathway-analysis).”

Supplementary Table S1. Primer sequences for RT-PCR used in this study.

Scn3a:
Forward: 5'-TCCGAGCCTTATCCCGCTTTGA-3',
Reverse: GAAGATGAGGCACACCAGTAGC;

Gabrg2:
Forward: 5'-ATCACCACTCCCAACAGGATGC-3',
Reverse: 5'-GCAGGAGTGTTCATCCATTGGG-3';

Kcnq3:
Forward: 5'-AAGCCTACGCTTTCTGGCAGAG-3',
Reverse: 5'-ACAGCTCGGATGGCAGCCTTTA-3';

Rny1:
Forward: 5'-CTGGTCCGAAGGTAGTGAGTTA-3',
Reverse: 5'-GTCAAGTGCAGTAGTGAGAAGG-3';

Postn:
Forward: 5'-CAGCAAACCACTTTCACCGACC-3';
Reverse: 5'-AGAAGGCGTTGGTCCATGCTCA-3';

Synpr:
Forward: 5'-CAGCCTTCCAACAAGTGCATGG-3',
Reverse: 5'-TCTCTGTCCAGAGGAATGCCAG-3';

Dscam:
Forward: 5'-CATCCGCATGTACGCCAAGAAC-3',
Reverse: 5'-GAGATGAGGTGGGTTCCAAGTG-3';

Sptbn2:
Forward: 5'-GTGGCAGAAACACCAGGCATTC-3',
Reverse: 5'-CTCCAGCTTCTCTGACACTACG-3';

Nfil3:
Forward: 5'-CAGGACTACCAGACATCCAAGG-3',
Reverse: 5'-AGGACACCTCTGACACATCGGA-3';

Kcns1:
Forward: CAAGGTAGTGCAAGTGTTCCGC,
Reverse: 5'-GTAAGATGCCCACCTCACGGTA-3';

As3mt:
Forward: 5'-TCCACGTTTGGTCACTGCCGAT-3',
Reverse: 5'-GAAGAGGCGAAATGTGGCAGAC-3'.

Supplementary Table S2. RNA-seq data stats. The sequencing information of the 18 RNA-seq samples with 6 conditions and 3 replicates.

Sample ID	Cell type	Inducer	Time point	Reads	Mappings	Reads pairs	Paired mappings	Paired properly mappings
D0_1	NSC (E14.5)	Control	Day 0	151987844	122806864	120554362	98251805	81.50%
D0_2	NSC (E14.5)	Control	Day 0	147847952	121866091	119456448	97954287	82.00%
D0_3	NSC (E14.5)	Control	Day 0	148745454	125661874	61810350	52600608	85.10%
D2Con_1	NSC (E14.5)	Control	Day 2	149498180	133972876	65936434	58815299	89.20%
D2Con_2	Diff NSC	Control	Day 2	157703498	140716726	69262464	61712855	89.10%
D2Con_3	Diff NSC)	Control	Day 2	174400960	156152788	76848844	68549169	89.20%
D8CN_1	Diff NSC	Control	Day 8	135734856	122874724	60726763	54957721	90.50%
D8CN_2	Diff NSC	Control	Day 8	140009768	125671100	62104707	55832132	89.90%
D8CN_3	Diff NSC	Control	Day 8	143367754	130406059	64476331	58608985	90.90%
D2BE_1	Diff NSC	Ethanol	Day 2	122424622	110684452	54702574	49341722	90.20%
D2BE_2	Diff NSC	Ethanol	Day 2	198252362	181338451	89666165	81775542	91.20%
D2BE_3	Diff NSC	Ethanol	Day 2	113258892	103647441	51252444	46690976	91.10%
D8CE_1	Diff NSC	Ethanol	Day 8	132558140	120229250	59451549	53863103	90.60%
D8CE_2	Diff NSC	Ethanol	Day 8	155767468	139754398	69059059	61876917	89.60%
D8CE_3	Diff NSC	Ethanol	Day 8	139379900	118226226	57986815	48766911	84.10%
D8WE_1	Diff NSC	Ethanol	Day 8 withdraw	141937290	123503144	60745419	52423297	86.30%
D8WE_2	Diff NSC	Ethanol	Day 8 withdraw	132438538	115684806	56954396	49493370	86.90%
D8WE_3	Diff NSC	Ethanol	Day 8 withdraw	179488604	154165822	75652780	64304863	85.00%
